# Metabolic Consequences of Polyphosphate Synthesis and Imminent Phosphate Limitation

**DOI:** 10.1128/mbio.00102-23

**Published:** 2023-04-19

**Authors:** Geun-Don Kim, Danye Qiu, Henning Jacob Jessen, Andreas Mayer

**Affiliations:** a Department of Immunobiology, University of Lausanne, Epalinges, Switzerland; b Institute of Organic Chemistry, University of Freiburg, Freiburg, Germany; University of British Columbia

**Keywords:** *Saccharomyces cerevisiae*, acidocalcisome, phosphate signalling, polyphosphate, SPX domains

## Abstract

Cells stabilize intracellular inorganic phosphate (P_i_) to compromise between large biosynthetic needs and detrimental bioenergetic effects of P_i_. P_i_ homeostasis in eukaryotes uses Syg1/Pho81/Xpr1 (SPX) domains, which are receptors for inositol pyrophosphates. We explored how polymerization and storage of P_i_ in acidocalcisome-like vacuoles supports Saccharomyces cerevisiae metabolism and how these cells recognize P_i_ scarcity. Whereas P_i_ starvation affects numerous metabolic pathways, beginning P_i_ scarcity affects few metabolites. These include inositol pyrophosphates and ATP, a low-affinity substrate for inositol pyrophosphate-synthesizing kinases. Declining ATP and inositol pyrophosphates may thus be indicators of impending P_i_ limitation. Actual P_i_ starvation triggers accumulation of the purine synthesis intermediate 5-aminoimidazole-4-carboxamide ribonucleotide (AICAR), which activates P_i_-dependent transcription factors. Cells lacking inorganic polyphosphate show P_i_ starvation features already under P_i_-replete conditions, suggesting that vacuolar polyphosphate supplies P_i_ for metabolism even when P_i_ is abundant. However, polyphosphate deficiency also generates unique metabolic changes that are not observed in starving wild-type cells. Polyphosphate in acidocalcisome-like vacuoles may hence be more than a global phosphate reserve and channel P_i_ to preferred cellular processes.

## INTRODUCTION

Inorganic phosphate (P_i_) is an essential nutrient and is a component of lipids and nucleic acids, controls the activity of proteins through covalent modification, and serves as an energy transducer when integrated into nucleotides, driving many endergonic biochemical reactions. Therefore, perturbed P_i_ homeostasis strongly affects growth and development of various living organisms (1–4). Eukaryotic cells have developed multilayered systems to maintain cytosolic P_i_ concentration within a suitable range despite fluctuating environmental conditions. Shortage of intracellular P_i_ was proposed to be signaled through a dedicated signaling pathway for intracellular phosphate reception and signaling (INPHORS), where the level of cytosolic P_i_ is translated into changes of inositol pyrophosphates (PP-IPs), which then bind to Syg1/Pho81/Xpr1 (SPX) receptor domains (5, 6). These domains form part of or interact with numerous cellular proteins that control transcription or mediate uptake, secretion, storage, and recycling of phosphate (7, 8). It is expected that INPHORS coordinates these systems such that cytosolic P_i_ concentration is maintained in a viable range.

In Saccharomyces cerevisiae, INPHORS triggers the PHO pathway, the transcriptional phosphate starvation response that controls expression of genes for P_i_ scavenging, uptake, and recycling (9). The PHO pathway in yeast is controlled through the transcription factor Pho4, which accumulates in the nucleus after P_i_ starvation and interacts with another transcription factor, Pho2, to activate many P_i_-dependent genes. The interaction between Pho2 and Pho4 is impaired by phosphorylation of Pho4 through Pho85/Pho80 kinase (10, 11). Early studies postulated that P_i_ starvation triggers an increase in 1-IP_7_, which should inactivate Pho85/Pho80 kinase by direct interaction with a central part of its regulatory subunit Pho81 (12, 13). However, several subsequent studies failed to confirm the starvation-induced increase in IP_7_ and instead showed a strong decline in the inositol phosphate pools after Pi starvation (5, 9, 14). These qualitatively different results on the inositol pyrophosphate pools and additional characterization of Pho81 led to a revised model of PHO pathway regulation in which 1,5-IP_8_ controls Pho81 through its SPX domain. The decline of 1,5-IP_8_, which is triggered by P_i_ starvation, allows Pho81 to bind Pho85/Pho80 more tightly and inhibit its kinase activity, favoring dephosphorylation and activation of Pho4. Pho4-dependent transcription depends on the interaction of Pho4 with Pho2, which is stimulated by Pho4 dephosphorylation (9–11). This interaction is also stimulated by an intermediate of purine synthesis, 5-aminoimidazole-4-carboxamide ribonucleotide (AICAR), but it is unknown whether AICAR levels respond to P_i_ availability (15–17).

Many organisms developed strategies to cope with a gradual decline (scarcity) or final absence (starvation) of phosphate, which then becomes growth limiting (1–4). Budding yeast induces its starvation response in at least two stages depending on the degree of P_i_ depletion (18–20). Phosphate scarcity activates P_i_ scavenging from the environment and maintains growth. Acid phosphatases, such as Pho5, are expressed and secreted to liberate P_i_ from external P_i_-containing molecules (21). As a further measure to facilitate P_i_ scavenging from the environment, low-affinity transporters are degraded and replaced by high-affinity transporters, such as Pho84 (22–25). Under complete P_i_ starvation, when P_i_ can no longer be obtained from the environment, yeast adopts different strategies. Cells recycle P_i_ by decomposing various intracellular organic molecules, including lipids, nucleotides, and even subcellular organelles (26–30). In addition, cells stop the cell cycle, saving P_i_ that would otherwise be used for nucleic acid and phospholipid duplication (31, 32).

In addition to scavenging and recycling, yeast cells also maintain a large store of phosphate inside their vacuoles (33, 34). These vacuoles share key features of acidocalcisomes, a class of conserved lysosome-like organelles that occur in all eukaryotic kingdoms (35, 36). These features include high lumenal concentrations of divalent cations, inorganic polyphosphates (poly[P]s), and basic amino acids. Their high poly(P) content is of potential relevance to phosphate homeostasis (18, 37, 38). This polymer can accumulate to the equivalent of hundreds of millimolar phosphate units in an osmotically almost inert form and is considered an efficient storage form of P_i_ (34, 39). Vacuolar poly(P) is produced from cytosolic ATP by the vacuolar transport chaperone (VTC) complex, which at the same time translocates the nascent poly(P) chain into the lumen of the organelle (40–42). Poly(P) can be hydrolyzed by polyphosphatases in the lumen of the organelle (43–46). It is assumed that the liberated P_i_ is released back into the cytosol to support metabolism. Therefore, poly(P) was proposed to act as a buffer system for cytosolic P_i_ (18, 47, 48). Synthesis of poly(P) by VTC is stimulated by inositol pyrophosphates (5, 49, 50). The levels of all these compounds (in yeast: 1-IP_7_, 5-IP_7_, and 1,5-IP_8_) increase in abundance with increasing P_i_ availability (9, 14) and will hence favor accumulation and storage of P_i_ in the form of poly(P) only if P_i_ is abundant. How the turnover of poly(P) inside the organelle is regulated and coordinated with the release of P_i_ into the cytosol is unknown.

To explore mechanisms that contribute to an early recognition of forthcoming P_i_ limitation, we explored the metabolic consequences experienced by yeast cells that are at the brink of phosphate limitation, that is, where P_i_ becomes scarce but not yet limiting for growth. Furthermore, we analyzed the metabolic significance of acidocalcisome-like vacuoles and their poly(P) pool.

## RESULTS

### Optimization of P_i_ starvation conditions for metabolomic analysis.

**FIG 1:**
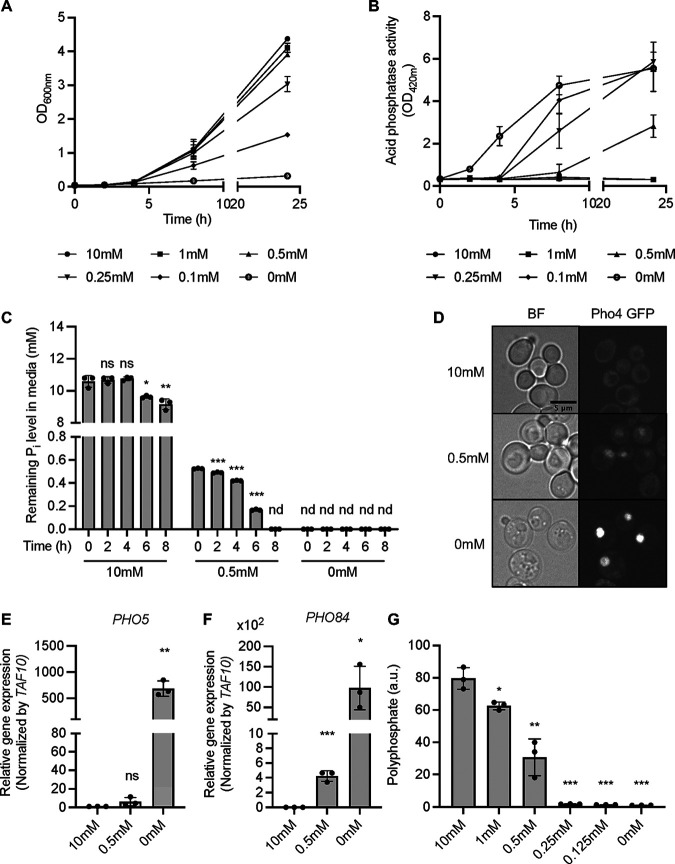
Response of S. cerevisiae under different P_i_ starving conditions. (A) Growth curves of yeast cells in synthetic complete medium supplemented with different concentrations of P_i_ from 10 mM to 0 mM. Cells were inoculated at an OD_600_ of 0.05 and cultured for 24 h. The means of triplicates are shown with standard deviation. (B) Acid phosphatase activities of yeast cells grown as in A. The means of triplicates are shown with standard deviation. (C) Concentrations of remaining P_i_ in the medium during cell growth. P_i_ concentration in the medium was monitored every 2 h for 8 h using the malachite green assay. The means of triplicates are shown with standard deviation; ***, *P* < 0.001; **, *P* < 0.01; *, *P* < 0.05; ns, not significant by Student’s *t* test; nd, not detected. (D) Fluorescence microscopy of live yeast cells producing Pho4 genomically tagged with GFP as the sole source of this protein. Cells were incubated for 8 h in 10 mM, 0.5 mM, and 0 mM P_i_ medium as in A before observation. (E and F) Relative gene expression levels of *PHO5* (E) and *PHO84* (F). Cells were grown in 10 mM, 0.5 mM, and 0 mM P_i_ medium for 8 h and harvested for RNA extraction and qRT-PCR. Fold change values were normalized with internal control *TAF10*. The means of three biological replicates are shown with standard deviation; ***, *P* < 0.001; **, *P* < 0.01; *, *P* < 0.05; ns, not significant by Student’s *t* test. (G) Polyphosphate levels in different P_i_-containing medium. Cells were incubated for 8 h as in A and harvested for polyphosphate measurement. The means of triplicates are shown with standard deviation; ***, *P* < 0.001; **, *P* < 0.01; *, *P* < 0.05 by Student’s *t* test; a.u., arbitrary units.

Yeast cells distinguish scarcity of P_i_ (where they induce genes to optimize P_i_ scavenging and maintain normal growth) from P_i_ starvation that reduces growth and induces genes to facilitate P_i_ recycling from internal sources (6, 18–20, 24, 51, 52). We used nontargeted metabolomic analysis to explore whether and how cells react to beginning P_i_ scarcity in comparison to profound P_i_ starvation. To this end, we first identified conditions that bring our strain background to the brink of P_i_ limitation of growth. Batch cultures of cells were grown logarithmically overnight until the optical density at 600 nm (OD_600_) was around 1 (4.6 × 10^7^ cells/mL). A small inoculum of these logarithmically growing cells was transferred into synthetic complete (SC) liquid medium containing 0 mM to 10 mM P_i_ (OD_600_ = 0.05; 2.3 × 10^6^ cells/mL), and the OD_600_ was followed over 24 h. Compared to cells growing in high-P_i_ medium (10 mM P_i_), growth was normal at 1 mM and 0.5 mM P_i_, and we observed only a mild retardation of growth in 0.25 mM and 0.1 mM P_i_. Incubation in 0 mM P_i_ arrested growth (Fig. 1A). We measured the activity of secreted acid phosphatase, which is activated by P_i_ starvation (21), as a readout of the PHO pathway. In P_i_-free medium, the activity of secreted acid phosphatase gradually increased up to 8 h and then maintained a similar level up to 24 h, suggesting that the PHO pathway was maximally activated at 8 h (Fig. 1B). In 0.1 mM and 0.25 mM P_i_, the activity of acid phosphatase was partially induced at 8 h but fully induced at 24 h, probably reflecting the gradual depletion of P_i_ from the medium over that period. In 0.5 mM P_i_ medium, acid phosphatase activity increased to half of the maximal value on 0 mM P_i_. There was no significant growth delay under this condition, indicating that the cells managed to compensate the limited availability of P_i_, probably through induction of the PHO pathway (Fig. 1A). To assess the P_i_ consumption of the cells under each condition, we monitored the amounts of P_i_ remaining in the medium using the malachite green assay (Fig. 1C). In 10 mM P_i_, more than 90% of P_i_ remained even after 8 h of incubation. In 0.5 mM P_i_ medium, 70% of the P_i_ was consumed after 6 h, and none remained after 8 h. We monitored this induction by tagging the Pho4 transcription factor with green fluorescent protein (GFP), which is translocated into the nucleus when cells lack P_i_. Pho4-GFP was predominantly in the nucleus after 8 h of incubation without P_i_ (Fig. 1D), and 0.5 mM P_i_ led to partial nuclear accumulation of Pho4-GFP, suggesting that the PHO pathway was moderately activated. We further measured the transcript level of the PHO pathway marker genes *Pho5* and *Pho84* using quantitative real-time reverse transcription-PCR (qRT-PCR). The gene expression of *Pho5* and *Pho84* was highly induced after 8 h of P_i_ starvation (Fig. 1E and F). Under P_i_ scarcity (0.5 mM P_i_), however, only *Pho84* showed a mild, yet statistically significant, induction, whereas induction of *Pho5* remained below 1% of the maximal value and was not significant. In agreement with earlier studies (18, 53), this suggests that partial induction of the PHO pathway allows the cells to compensate for reduced availability of P_i_ to a level that supports normal growth.

Under P_i_-limiting conditions, vacuolar poly(P) is degraded (18, 34, 45, 46, 54). It is assumed that resulting P_i_ is exported from the vacuoles to replenish the cytosolic P_i_ pool. In 0.25 mM, 0.125 mM, and 0 mM P_i_, poly(P) was completely degraded (Fig. 1G). However, after 8 h of growth in 0.5 mM P_i_, cells retained 40% of poly(P) compared to P_i_-replete conditions. We hence chose the described scheme of 8 h of growth in 0.5 mM P_i_ as a condition for metabolomic analysis under P_i_ limitation because it partially activates the PHO pathway, has no significant effect on cell growth, and allows partial maintenance of the vacuolar poly(P) pool.

### Complete P_i_ starvation induces broad metabolic changes.

**FIG 2:**
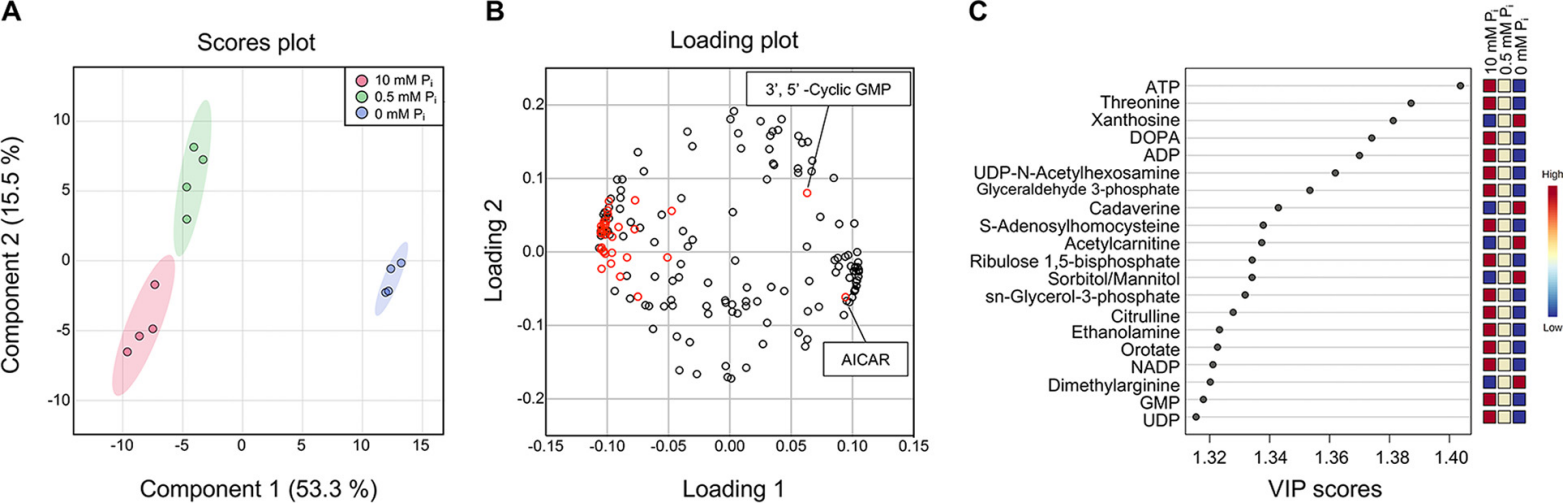
Partial least-squares discriminant analysis (PLS-DA) of S. cerevisiae metabolites under different P_i_ conditions. (A) Score plot of PLS-DA. Red, green, and blue dots indicate the replicates of yeast metabolomic data incubated in 10 mM, 0.5 mM, and 0 mM P_i_ medium, respectively. The shaded regions represent the 95% confidence intervals. (B) Loading plot of PLS-DA. Red dots indicate P_i_-containing metabolites. (C) Variable of importance in projection (VIP) scores of the top 20 metabolites generated from PLS-DA. The color code indicates the relative abundance of each metabolite under different P_i_ conditions.

For metabolome analyses, logarithmically growing cells were transferred into medium providing abundant P_i_ (10 mM), P_i_ scarcity (0.5 mM), or P_i_ starvation (0 mM P_i_). After 8 h of incubation in these medium formulations, 0.5 OD_600_ units of cells were harvested using vacuum filtration through a polytetrafluoroethylene polymer (PTFE) membrane and immediately frozen in liquid nitrogen (55). To analyze the metabolic effects of P_i_ limitation, untargeted metabolomic analyses were conducted through hydrophilic interaction liquid chromatography (LC)-mass spectrometry (HILIC-MS). A total of 169 metabolites were identified. Individual metabolic features were normalized by the median of each sample, transformed to log_10_, and centralized to the mean by an autoscaling method for further statistical analyses. Partial least-squares discriminate analysis (PLS-DA) was performed to condense the metabolomic data into a simple plot, allowing easy comparison of overall metabolic features (Fig. 2A). This revealed a clear separation of the three growth conditions. Component 1, which comprises the largest difference of the total variance in metabolites (53.3%), placed the 0 mM P_i_ samples far from 10 mM and 0.5 mM P_i_ samples, suggesting that the metabolic changes caused by P_i_ starvation were greater than by P_i_ limitation (Fig. 2A). In the loading plot, which visualizes the contribution of individual metabolites to components 1 and 2, most phosphate-containing metabolites (red open circle) negatively contributed to component 1, indicating that they decreased under P_i_ starvation (Fig. 2B). Only two phosphate-containing metabolites, AICAR and 3′,5′-cyclic GMP (3′,5′-cGMP), positively contributed to component 1. For further analysis, we compared variable importance in projection (VIP) scores of each metabolite, which represent the contribution of variables to the PLS-DA model encompassing 10 mM, 0.5 mM, and 0 mM P_i_ data. ATP showed the highest VIP value among the top 20 most influential metabolites (Fig. 2C), underscoring an interrelation of P_i_ and ATP metabolism (56).

**FIG 3:**
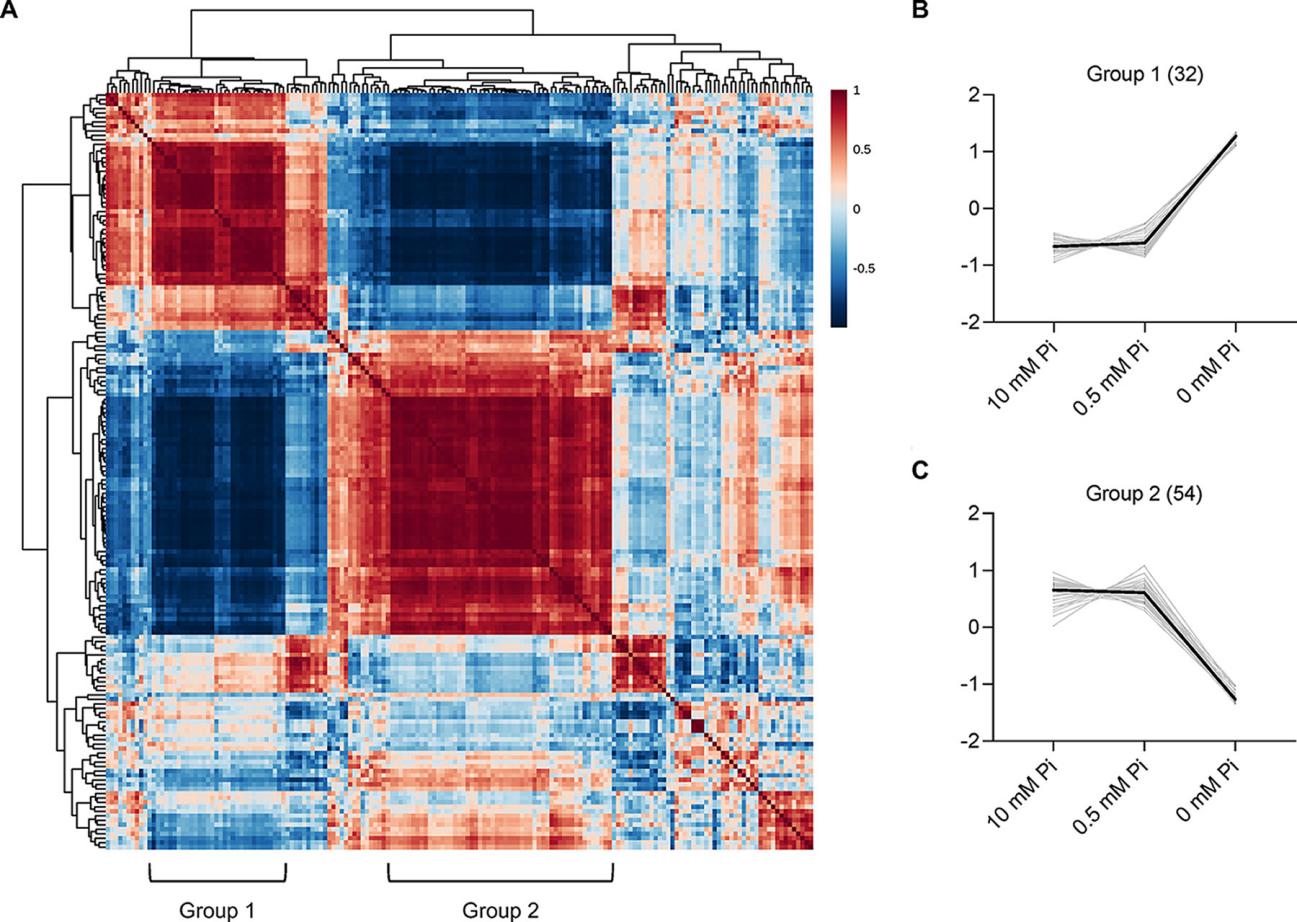
Correlation analysis of S. cerevisiae metabolites under different P_i_ starving conditions. (A) Clustered correlation heatmap between metabolites under different P_i_ conditions. The correlation matrix was generated by Pearson correlation coefficients, which are represented by a color code. Red and blue indicate positive and negative correlations, respectively. Two representative metabolic groups showing strong correlations are marked as group 1 and group 2. (B and C) Relative abundance of metabolites included in group 1 (B) and group 2 (C) under different P_i_ conditions. The profiles of individual metabolites are shown in gray.

Pearson coefficients were calculated as a measure for the correlation between metabolites. Two metabolic groups (group 1 and group 2) were negatively correlated with each other (Fig. 3A). The relative abundance of metabolites in 0 mM P_i_ medium increased for group 1 and decreased for group 2 (Fig. 3B and C). Purine and pyrimidine pathway metabolites, nucleosides, and nucleobases increased (Table 1), mirrored by a decrease of nucleotides. Metabolites of the citrate cycle (TCA) increased, such as citric acid, isocitric acid, and oxoglutaric acid, whereas the levels of glycolytic intermediates declined, suggesting an altered strategy for energy production under P_i_ starvation. Nicotinate and nicotinamide metabolites also decreased. By contrast, metabolites of the tryptophan degradation pathway, which are involved in NAD *de novo* synthesis, accumulated.

**TABLE 1:** List of metabolites in group 1 and group 2 from the correlation heatmap

Group	Metabolic pathway	Metabolites
Group 1	Purine	Deoxyadenosine, adenosine, AICAR, guanosine, inosine, xanthine, xanthosine
	Pyrimidine	Cytosine, uridine
	TCA	Citric acid, isocitric acid, oxoglutaric acid
	Cysteine and methionine	*S*-Adenosylmethionine, 5′-methylthioadenosine
	Tryptophan	Kynurenine, kynurenic acid, xanthurenic acid, quinolinic acid
	Glycerolipid	Choline, glyceric acid
	Others	5′-Deoxyadenosine, acetylcarnitine, cadaverine, glutamine, methylglutaric acid, *N*-acetylphenylalanine, *N*-methylglutamic acid, *N*-6-trimethyllysine, *p*-hydroxyphenylacetic acid, pantothenic acid, pyridoxal
Group 2	Purine	AMP, ADP, ADP ribose, ATP, dATP, GMP, GDP, guanine, IMP, hypoxanthine
	Pyrimidine	CMP, CDP, dCDP, UMP, UDP, orotic acid
	Glycolysis	Acetyl-CoA, fructose-6-phosphate, glyceraldehyde-3-phosphate, glucose-6-phosphate, 2/3-phosphoglyceric acid, phosphoenolpyruvic acid
	Cysteine and methionine	1-Aminocyclopropanecarboxylic acid, ophthalmic acid, *S*-adenosylhomocysteine, cystathionine, 3-sulfinoalanine, pyroglutamic acid
	Nicotinate and nicotinamide	NAD^+^, NADP^+^, nicotinic acid mononucleotide
	Glycerolipid	*sn*-Glycerol-3-phosphate, ethanolamine, glycerol
	Arginine	*N*-Acetylputrescine, proline, *N*-acetylglutamic acid, citrulline, dimethylarginine
	Others	Guaiacol, aminoisobutyric acid, 2-hydroxyglutaric acid, aspartic acid, glutamic acid, glycine, histidine, lysine, serine, threonine, mevalonic acid, *N*^2^-acetyllysine, TMP, UDP-hexose, UDP-*N*-acetylhexosamine

### Pathway analysis of P_i_ starvation.

**FIG 4:**
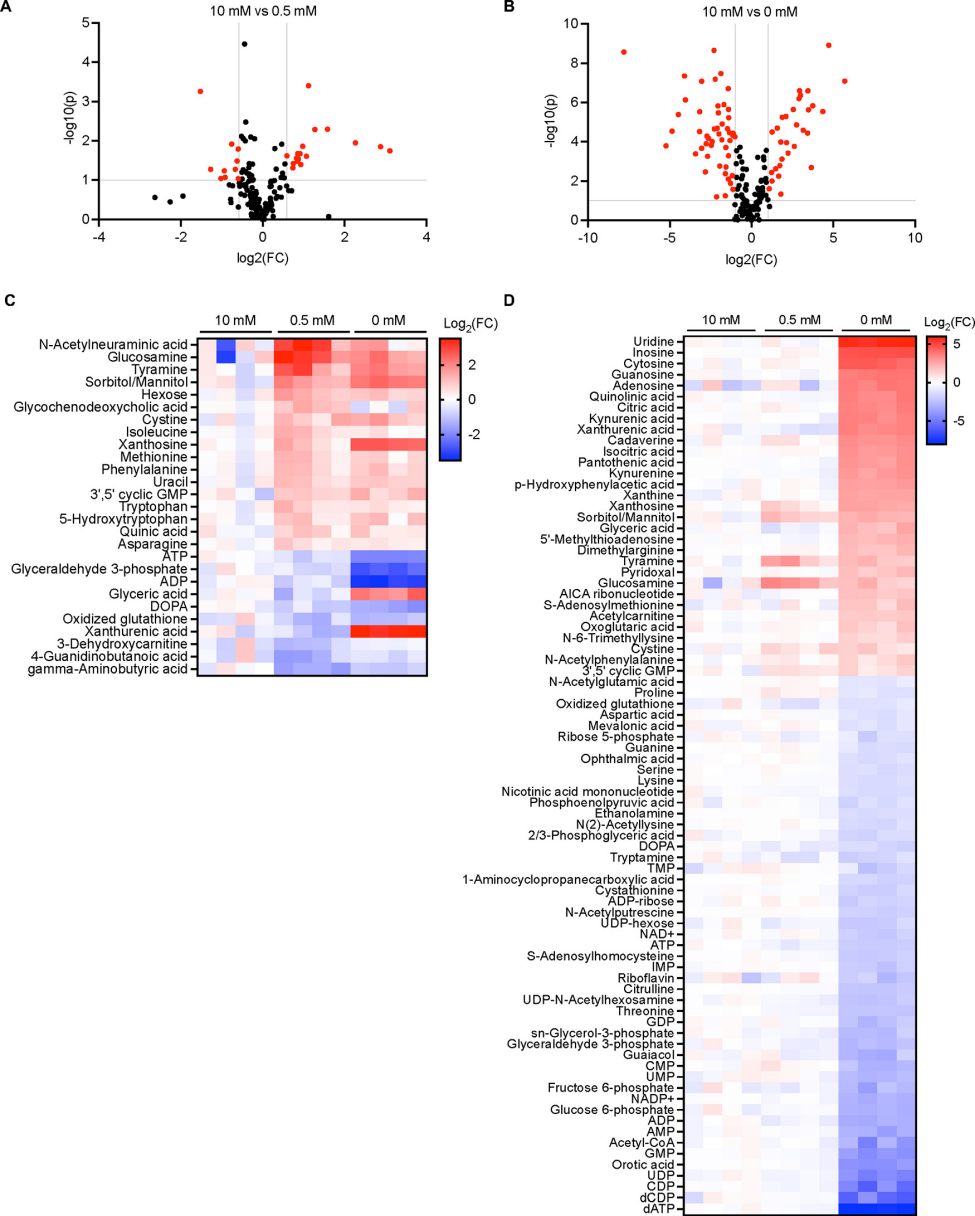
Metabolites differentially accumulated under P_i_ limitation or P_i_ starvation. Volcano plot analysis of changes after P_i_ limitation and P_i_ starvation. (A) Changes after P_i_ limitation (0.5 mM Pi). Red dots indicate differentially accumulated metabolites (fold change, |FC| > 1.5; *P* < 0.1). (B) Changes after P_i_ starvation (0 mM P_i_). Red dots indicate differentially accumulated metabolites (fold change, |FC| > 2; *P* < 0.1). These metabolites are listed in Data Set S1. (C) Heatmap of P_i_ limitation. The list was selected by *t* test (*P* < 0.1), showing metabolites changing at least 1.5-fold under P_i_ limitation (0.5 mM P_i_). (D) Heatmap of P_i_ starvation. Same as in C but shows metabolites changing at least 2-fold under P_i_ starvation (0 mM P_i_). The relative abundance of metabolites is represented as log_2_ (fold change) through a color code.

**FIG 5:**
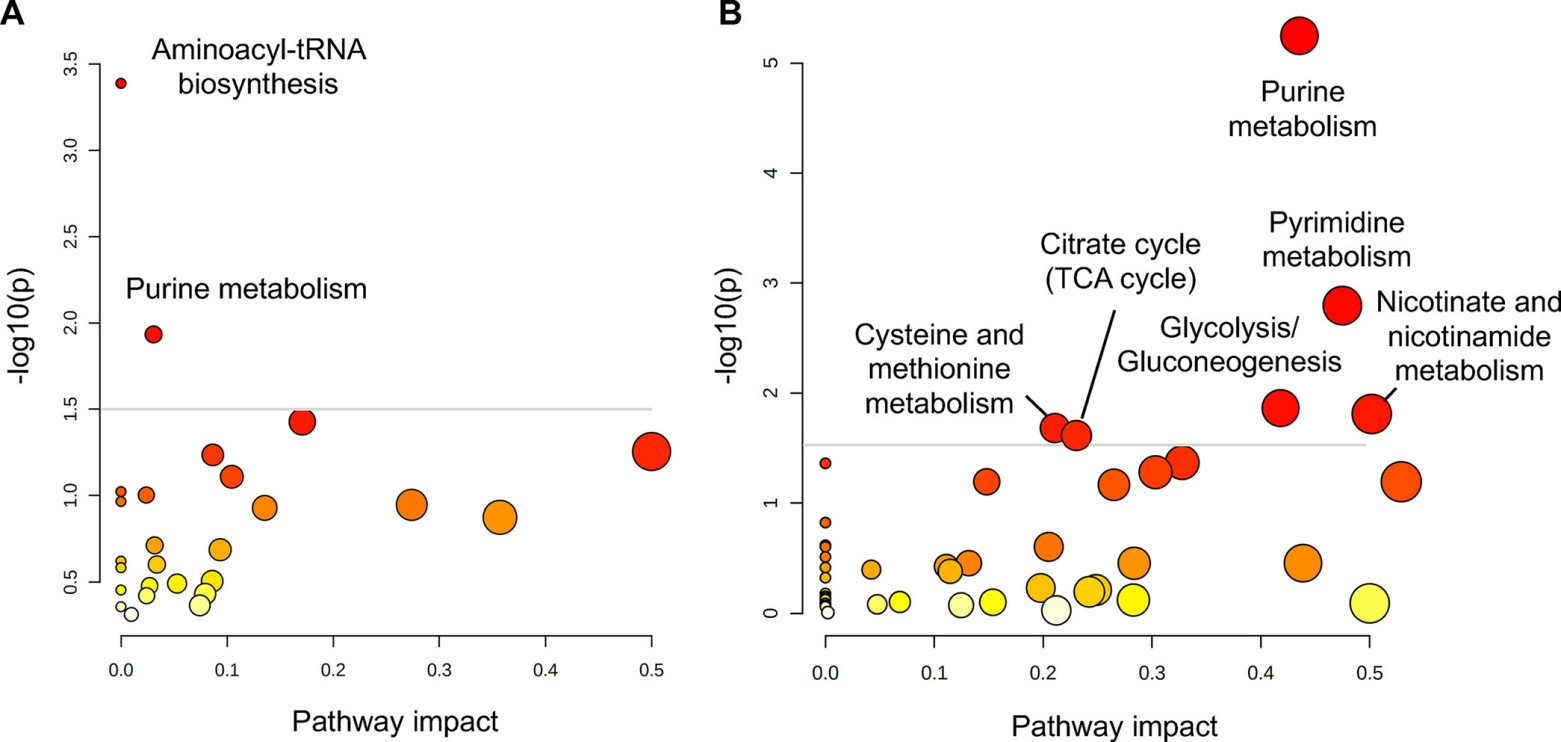
Pathway analysis of differentially accumulated metabolites under different P_i_ starving conditions. (A and B) Pathway analysis of differentially accumulated metabolites after P_i_ limitation (0.5 mM P_i_) (A) and P_i_ starvation (0 mM P_i_) (B). The size and color of the circle indicate impact value and *P* value, respectively. The annotated metabolic pathways have higher statistical significance (−log [*P*] > 1.5).

To identify metabolites that accumulated differentially and in a statistically significant manner, we compared their relative abundance by volcano plots. Profound metabolic effects occurred under complete P_i_ starvation; 31 metabolites increased and 49 decreased in a statistically meaningful way, representing 47% of the detected metabolites (Fig. 4B and D; Data Set S1 in the supplemental material). Pathway analysis of these 80 metabolites identified the most affected pathways as purine metabolism, pyrimidine metabolism, nicotinate and nicotinamide metabolism, glycolysis/gluconeogenesis, citrate cycle, and cysteine and methionine metabolism, with a −log (*P* value) of >1.5 (Fig. 5B; Table 2).

**TABLE 2:** Six out of the top 15 metabolic sets are significantly affected under P_i_ starvation

Pathway^*a*^	Match status^*b*^	*P* value	−log (*P*)^*c*^	Impact^*d*^
**Purine metabolism**	**17/62**	**0.0000056885**	**5.245**	**0.43543**
**Pyrimidine metabolism**	**9/34**	**0.0016028**	**2.7951**	**0.47488**
**Glycolysis or gluconeogenesis**	**6/24**	**0.013697**	**1.8634**	**0.41811**
**Nicotinate and nicotinamide metabolism**	**4/12**	**0.015489**	**1.81**	**0.50185**
**Cysteine and methionine metabolism**	**8/41**	**0.02079**	**1.6822**	**0.21078**
**Citrate cycle (TCA cycle)**	**5/20**	**0.024305**	**1.6143**	**0.2306**
Methane metabolism	5/23	0.042866	1.3679	0.32774
Lysine biosynthesis	4/16	0.043511	1.3614	0.0
Glycine, serine, and threonine metabolism	6/32	0.052435	1.2804	0.30324
Arginine biosynthesis	4/18	0.064018	1.1937	0.14819
Pentose phosphate pathway	4/18	0.064018	1.1937	0.52896
Glyoxylate and dicarboxylate metabolism	5/26	0.068182	1.1663	0.26506
Cyanoamino acid metabolism	2/8	0.14937	0.82572	0.0
Synthesis and degradation of ketone bodies	1/3	0.23995	0.61987	0.0
Beta-alanine metabolism	2/11	0.24839	0.60487	0.0

a The data correspond to Fig. 4B; bold font indicates significant pathways.

b (Number of significant metabolites)/(total number of metabolites) in given pathway.

c The top 15 metabolic pathways were ranked based on *P* value. The metabolic pathways with a −log (*P*) value of >1.5 were considered significantly altered in this analysis.

d The impact value was calculated by pathway topology analysis.

10.1128/mbio.00102-23.9 DATA SET S1 Metabolites significantly affected by different Pi conditions. Download Data Set S1, XLSX file, 0.01 MB. Copyright © 2023 Kim et al. 2023 Kim et al. https://creativecommons.org/licenses/by/4.0/ This content is distributed under the terms of the Creative Commons Attribution 4.0 International license.

10.1128/mbio.00102-23.10 DATA SET S2 Results of two-way ANOVA of wild-type and Δ*vtc4* cells under 2 h of P_i_-replete (10 mM P_i_) and P_i_-starving conditions (0 mM P_i_). Download Data Set S2, XLSX file, 0.01 MB. Copyright © 2023 Kim et al. 2023 Kim et al. https://creativecommons.org/licenses/by/4.0/ This content is distributed under the terms of the Creative Commons Attribution 4.0 International license.

**(i) Glycolysis and the TCA cycle.** P_i_ starvation reduced the abundance of glycolysis intermediates 2- to 10-fold, including glucose-6-phosphate, fructose-6-phosphate, glyceraldehyde-3-phosphate, 2/3-phosphoglyceric acid, phosphoenolpyruvic acid, and acetyl-coenzyme A (acetyl-CoA) (Fig. S1A). In addition, ribose-5-phosphate, which is derived from glucose-6-phosphate via the oxidative pentose phosphate pathway (PPP), was reduced. The upstream TCA cycle metabolites, including citric acid, isocitric acid, and oxoglutaric acid, accumulated 2- to 10-fold, whereas later TCA cycle metabolites, including succinic acid and malic acid, did not change (Fig. S1B). Thus, P_i_ starvation has a strong impact on glycolysis and the TCA cycle.

10.1128/mbio.00102-23.1 FIG S1 Metabolic changes in energy metabolism pathways under different P_i_ conditions. (A and B) Metabolomic data of yeast under different P_i_ conditions are presented on energy metabolic pathways, including glycolysis (A) and the TCA cycle (B). P_i_-containing substrates are marked as red. Gray indicates enzymes involved in the reaction. Different letters on the graph indicate a significant difference by Tukey-Kramer test (*P* < 0.05). Download FIG S1, TIF file, 0.7 MB. Copyright © 2023 Kim et al. 2023 Kim et al. https://creativecommons.org/licenses/by/4.0/ This content is distributed under the terms of the Creative Commons Attribution 4.0 International license.

**(ii) Nicotinate and nicotinamide metabolism.** The P_i_-containing metabolites nicotinic acid mononucleotide, NAD^+^, and NADP^+^ decreased 2- to 5-fold under P_i_ starvation (Fig. S2). By contrast, metabolites of NAD^+^ *de novo* synthesis, also known as the kynurenine pathway, accumulated more than 6-fold. Based on these results, it can be speculated that the impaired NAD^+^ synthesis from the kynurenine pathway will affect intracellular redox homeostasis.

10.1128/mbio.00102-23.2 FIG S2 Metabolic changes in nicotinate and nicotinamide pathway under different P_i_ conditions. Metabolomic data of yeast under different P_i_ conditions are presented on a metabolic pathway. P_i_-containing substrates are marked as red. Gray indicates enzymes involved in the reaction. Different letters on the graph indicate a significant difference by Tukey-Kramer test (*P* < 0.05). Download FIG S2, TIF file, 0.8 MB. Copyright © 2023 Kim et al. 2023 Kim et al. https://creativecommons.org/licenses/by/4.0/ This content is distributed under the terms of the Creative Commons Attribution 4.0 International license.

**(iii) Cysteine and methionine metabolism.** *S*-Adenosylmethionine (SAM) is the major methyl donor for modifications of various biomolecules, including proteins, DNA, RNA, and metabolites, producing *S*-adenosylhomocysteine (SAH) as a byproduct of the reaction. The relative abundance of SAM and of methionine salvage pathway metabolites increased while that of SAH decreased under P_i_ starvation (Fig. S3). Cystathionine, which can be produced from SAH via homocysteine, diminished under P_i_ starvation, whereas metabolites derived from cystathionine, such as glutathione and taurine, were not significantly affected. P_i_ starvation also affected another SAM-dependent branch, the synthesis of polyamines, because a byproduct of polyamine synthesis, 5′-methylthioadenosine (MTA), accumulated strongly. These results suggest that yeast cells change their strategy of SAM utilization under P_i_ deprivation. The reduction of nucleic acid synthesis, which accompanies growth arrest, may reduce consumption of SAM for nucleotide synthesis and promote accumulation of this compound.

10.1128/mbio.00102-23.3 FIG S3 Metabolic changes in methionine and cysteine metabolism pathways under different P_i_ conditions. Metabolomic data of yeast under different P_i_ conditions are presented on a metabolic pathway. P_i_-containing substrates are marked as red. Gray indicates enzymes involved in the reaction. Different letters on the graph indicate a significant difference by Tukey-Kramer test (*P* < 0.05). Download FIG S3, TIF file, 0.8 MB. Copyright © 2023 Kim et al. 2023 Kim et al. https://creativecommons.org/licenses/by/4.0/ This content is distributed under the terms of the Creative Commons Attribution 4.0 International license.

**(iv) Purine and pyrimidine metabolism.** Nucleosides and nucleobases, such as adenosine, inosine, xanthosine, guanosine, uridine, cytosine, and xanthine, significantly accumulated under P_i_ starvation (Fig. S4A and B), whereas P_i_-containing nucleotides, nucleoside diphosphates, and nucleoside triphosphates decreased. By contrast, the amount of AICAR increased. This metabolic change may contribute to triggering the PHO pathway under P_i_ starvation in two ways. First, AICAR inhibits the production of IP_8_ (57), which itself is a potent suppressor of the PHO pathway (9). Second, AICAR stabilizes the interaction of the association of the transcription factors Pho4 and Pho2, which is necessary for full induction of the PHO pathway (17).

10.1128/mbio.00102-23.4 FIG S4 Metabolic changes in purine and pyrimidine metabolism pathways under different P_i_ conditions. (A and B) Metabolomic data of yeast under different P_i_ conditions are presented on a purine metabolic pathway (A) and a pyrimidine metabolic pathway (B). P_i_-containing substrates are marked as red. Gray indicates enzymes involved in the reaction. Different letters on the graph indicate a significant difference by Tukey-Kramer test (*P* < 0.05). Download FIG S4, TIF file, 0.8 MB. Copyright © 2023 Kim et al. 2023 Kim et al. https://creativecommons.org/licenses/by/4.0/ This content is distributed under the terms of the Creative Commons Attribution 4.0 International license.

### Moderate P_i_ limitation causes few potentially diagnostic metabolic changes.

**FIG 6:**
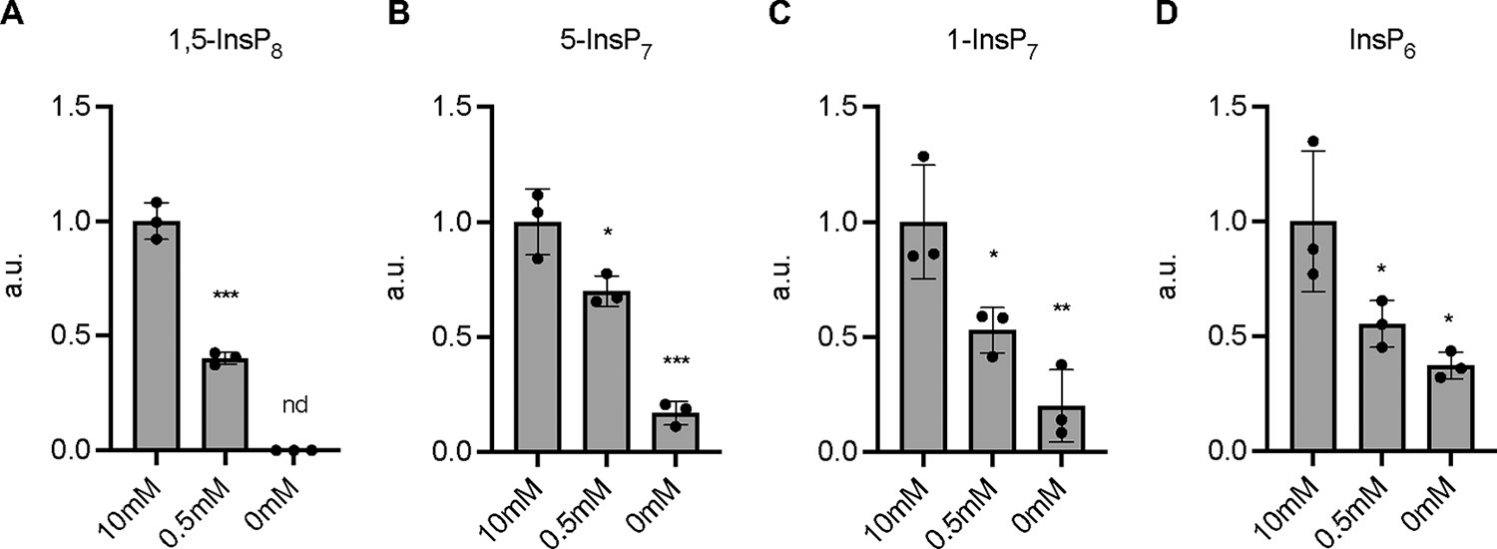
Inositol pyrophosphate profiles under different P_i_ conditions. (A to D) Inositol pyrophosphate levels of yeast cells grown in 10 mM, 0.5 mM, and 0 mM P_i_ medium for 8 h. The data were normalized by the number of cells. The amount of each inositol pyrophosphate in 10 mM P_i_ medium was set to 1. The means of triplicates are shown with standard deviation; ***, *P* < 0.001; **, *P* < 0.01; *, *P* < 0.05 by Student’s *t* test; nd, not detected; a.u., arbitrary unit.

Few significant changes (−log [*P*] > 1) occurred under P_i_-limiting conditions; only 17 metabolites increased and 10 decreased more than 1.5-fold (Fig. 4A and C and 5A; Data Set S1). ATP decreased in a statistically significant manner, which suggests that the ATP level is more sensitive to P_i_ availability than most other metabolites, making ATP a bona fide early indicator of P_i_ scarcity. In line with this, one class of key enzymes for signaling the intracellular P_i_ state, IP_6_ kinases, have an unusually high *K_m_* for ATP, which is close to the normal cellular concentration of this compound (58). To test this possibility, we measured the products of these enzymes, inositol pyrophosphates, under different P_i_ conditions using capillary electrophoresis-coupled electrospray ionization mass spectrometry (CE-ESI-MS). Under P_i_ starvation, 1,5-IP_8_ was not detected at all, 5-IP_7_ and 1-IP_7_ decreased by 80%, and IP_6_ decreased by 60% (Fig. 6A to D). In contrast to the PP-IPs, the decrease of IP_6_ occurred only with a lag phase of 2 h (Fig. S8A to D). Even under mild P_i_ scarcity, all four compounds significantly declined in comparison with P_i_-replete conditions, by 60% for 1,5-IP_8_, 30% for 5-IP_7_, and 50% for 1-IP_7_ and IP_6_. This is consistent with the hypothesis that even moderate decreases in ATP levels under P_i_ limitation can be translated into decreased PP-IP levels, which then activate SPX domain-based signaling to stabilize cytosolic P_i_.

10.1128/mbio.00102-23.8 FIG S8 Inositol pyrophosphate profiles. Time course analysis under P_i_ starvation and Δ*vtc4* under P_i_-replete conditions. (A to D) Time course of PP-IP levels of wild-type yeast cells transferred to 0 mM P_i_ medium for 8 h. Logarithmically growing yeast cells in 10 mM P_i_ medium were transferred into 0 mM P_i_ medium. In contrast to the direct extraction of the cultures with perchloric acid in Fig. 6, cells were recovered by filtration after the indicated periods of P_i_ starvation. The filters were extracted with perchloric acid, and the inositol pyrophosphates were quantified by CE-MS. Note that the filtration method used here is less efficient for the small quantities of remaining PP-IPs after starvation. The data were normalized by the number of cells per sample. The concentration of each inositol pyrophosphate in 10 mM P_i_ medium was set to 1. (E to H) Wild-type and Δ*vtc4* yeast cells were logarithmically grown in 10 mM P_i_ medium overnight, harvested by centrifugation, and subjected to perchloric acid extraction and PP-IP analysis as in A. The data were normalized by the number of cells. The amount of each inositol pyrophosphate in wild-type cells was set to 1. Means with standard deviations are shown; *n* = 3; a.u., arbitrary units; ***, *P* < 0.001; **, *P* < 0.01; *, *P* < 0.05; ns, not significant by Student’s *t* test. Download FIG S8, TIF file, 0.5 MB. Copyright © 2023 Kim et al. 2023 Kim et al. https://creativecommons.org/licenses/by/4.0/ This content is distributed under the terms of the Creative Commons Attribution 4.0 International license.

### Poly(P) in acidocalcisome-like vacuoles contributes to P_i_ homeostasis even under P_i_-replete conditions.

**FIG 7:**
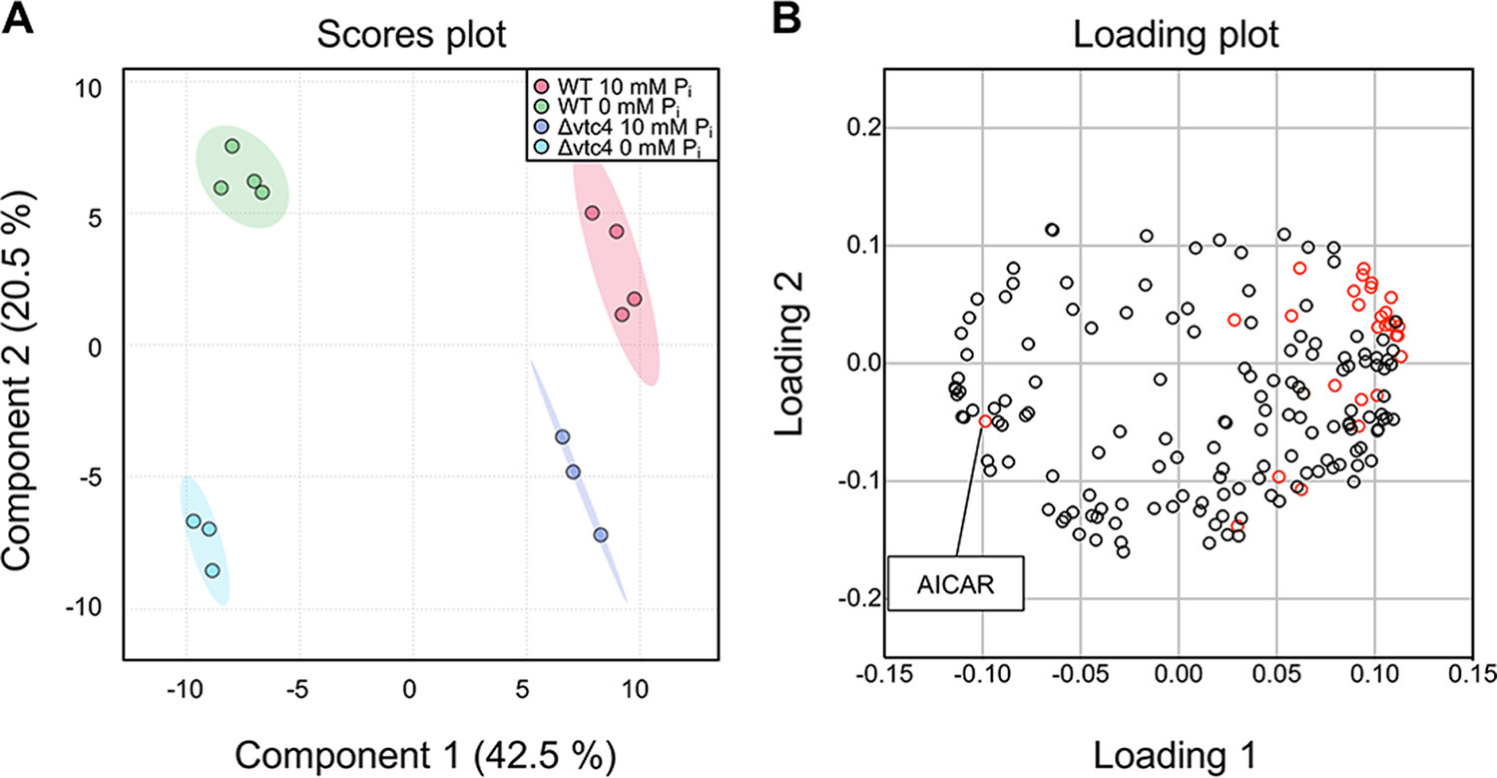
Multivariate statistical analysis of metabolite profiling data from wild-type and Δ*vtc4* cells under 10 mM and 0 mM P_i_ conditions. (A) Score plot of partial least-squares discriminant analysis (PLS-DA). Red, green, blue, and light blue indicate the replicates of metabolomic data from wild-type 10 mM P_i_, wild-type 0 mM P_i_, Δ*vtc4* 10 mM P_i_, and Δ*vtc4* 0 mM P_i_, respectively. The shaded regions represent the 95% confidence intervals. (B) Loading plot of PLS-DA. Red dots indicate P_i_-containing metabolites.

Yeast cells contain acidocalcisome-like vacuoles, which can convert the γ-phosphate from ATP into inorganic polyphosphate. Thereby, they can store phosphate units at concentrations in the hundreds of millimolar in an osmotically inactive form (40, 41). At the same time, vacuoles contain polyphosphatases and P_i_ exporters, which can hydrolyze poly(P) and export the liberated P_i_ to the cytosol (46). This system is powerful enough to influence P_i_ homeostasis of the cells and the P_i_ starvation response when it is dysregulated (18, 37, 38, 54). To investigate the metabolic role of vacuolar polyphosphates, we analyzed the metabolic profiles of the Δ*vtc4* mutant, which lacks the poly(P)-synthesizing complex VTC, under P_i_-replete conditions and P_i_ starvation. To maximize the chance of observing poly(P)-dependent differences, we restricted the starvation period to 2 h because wild-type cells mobilize their poly(P) pool over the first 2 to 3 h of P_i_ starvation (18–20). A PLS-DA plot showed that the metabolic features of Δ*vtc4* cells were clearly distinct from those of the wild type under P_i_-rich conditions and even more under P_i_ starvation (Fig. 7A). The loading plot of PLS-DA revealed that almost all phosphate-containing metabolites (red open circles) were abundant under P_i_-rich conditions, except AICAR, consistent with the results above (Fig. 2B and 7B).

**FIG 8:**
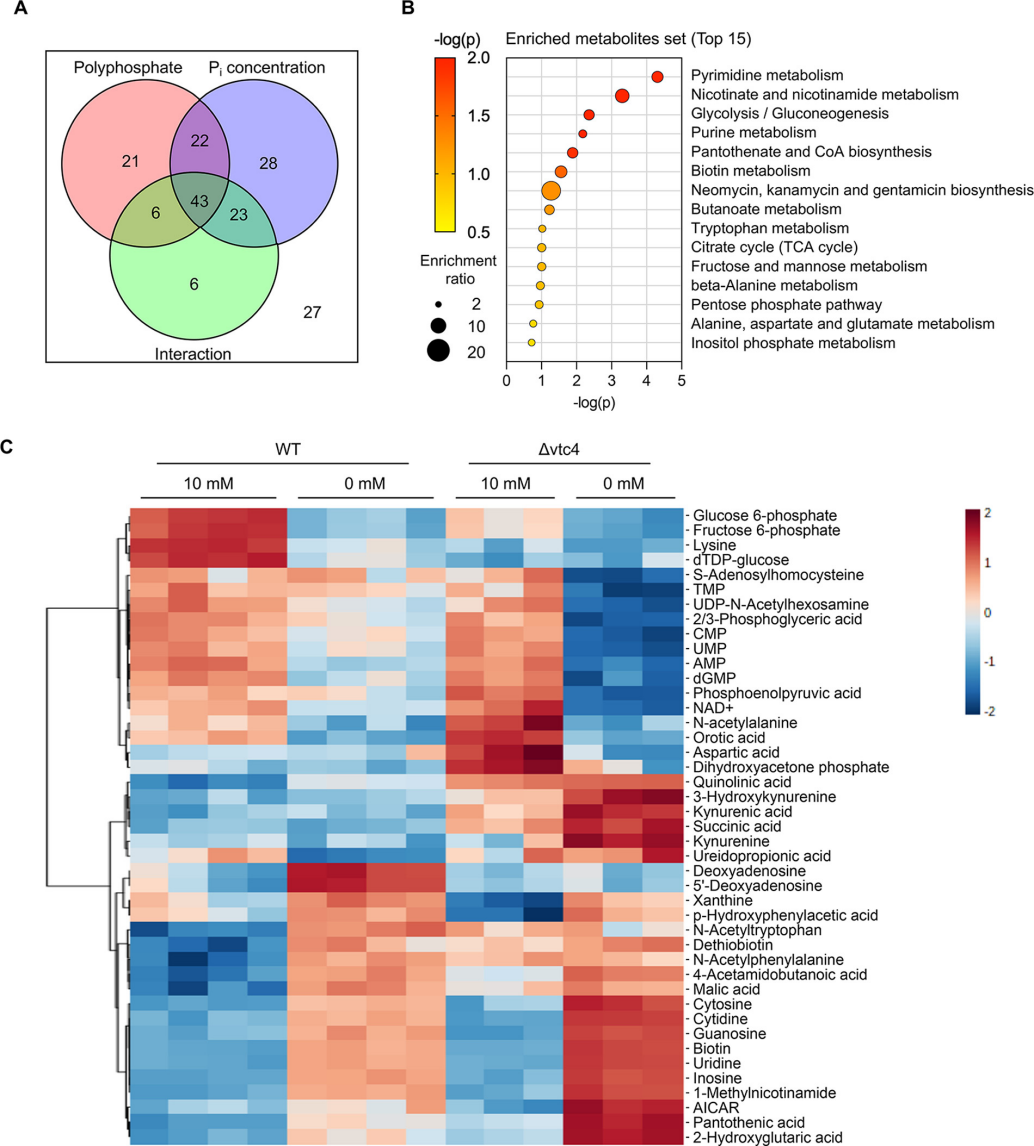
Interrelated effect of polyphosphate and P_i_ starvation on metabolic pathways. (A) Summary of two-way ANOVA analysis (adjusted *P* value of <0.05). Red, blue, and green represent the metabolites affected by polyphosphate (wild type and Δ*vtc4*), P_i_ concentration (10 mM and 0 mM P_i_), and interaction between both (polyphosphate and P_i_ concentration), respectively. (B) Metabolite set enrichment analysis of 43 metabolites simultaneously affected by polyphosphate, P_i_ concentration, and their interaction. The top 15 metabolite sets were selected based on *P* value. (C) A heatmap was generated based on the list of 43 metabolites affected by P_i_ concentration, poly(P), and their interaction from a two-way ANOVA. Features were clustered by Euclidean distance using Ward’s clustering method. The color code indicates the normalized intensity of metabolic features.

To analyze how polyphosphate synthesis and P_i_ concentration in the medium affect metabolic features, a two-way analysis of variance (ANOVA) was performed. The relative abundances of 92, 116, and 78 metabolic features were affected by poly(P), P_i_ concentration, and their interaction, respectively (Fig. 8A; Data Set S2). A total of 65 metabolites were affected by both poly(P) and P_i_, of which 66% (43 features) were additionally affected by their interaction. Metabolite set enrichment analysis was performed using these 43 metabolites. Most metabolic pathways affected by P_i_ starvation shown in the previous pathway analysis, such as pyrimidine metabolism, nicotinate and nicotinamide metabolism, glycolysis, and purine metabolites, were again ranked statistically high, showing consistency between the analyses (Fig. 8B; Table 3). The changes of these 43 metabolites were visualized by heatmap analysis (Fig. 8C). Δ*vtc4* cells showed much more pronounced changes than wild-type cells in several respects, including the decrease of P_i_-containing purines and pyrimidines (CMP, UMP, AMP, and dGMP) (Fig. 8C; Fig. S5A and B), the increase of nucleosides and nucleobases (cytosine, cytidine, guanosine, uridine, and inosine) (Fig. 8C; Fig. S7 and S8), and the reduction of NAD^+^ and NADP^+^ (Fig. S6).

**TABLE 3:** Six out of the top 15 pathways emerging from metabolite set enrichment analysis are significant

Metabolite set^*a*^	Total	Hits	Expected	*P* value^*b*^
**Pyrimidine metabolism**	**39**	**7**	**1.05**	**0.0000494**
**Nicotinate and nicotinamide metabolism**	**15**	**4**	**0.404**	**0.0005**
**Glycolysis/gluconeogenesis**	**26**	**4**	**0.7**	**0.00441**
**Purine metabolism**	**65**	**6**	**1.75**	**0.00666**
**Pantothenate and CoA biosynthesis**	**19**	**3**	**0.512**	**0.013**
**Biotin metabolism**	**10**	**2**	**0.269**	**0.0278**
Neomycin, kanamycin, and gentamicin biosynthesis	2	1	0.0539	0.0532
Butanoate metabolism	15	2	0.404	0.0596
Tryptophan metabolism	41	3	1.1	0.0954
Citrate cycle (TCA cycle)	20	2	0.539	0.0991
Fructose and mannose metabolism	20	2	0.539	0.0991
β-Alanine metabolism	21	2	0.566	0.108
Pentose phosphate pathway	22	2	0.593	0.117
Alanine, aspartate, and glutamate metabolism	28	2	0.754	0.173
Inositol phosphate metabolism	30	2	0.808	0.192

a The data correspond to Fig. 8B; bold font indicates significant pathways.

b The top 15 metabolite sets were ranked based on *P* value. Metabolic sets with a *P* value of <0.05 were considered significantly altered.

10.1128/mbio.00102-23.5 FIG S5 Metabolic changes in purine and pyrimidine metabolism pathways under P_i_ starvation in wild-type and Δ*vtc4* cells. (A and B) Metabolomic data of yeast under P_i_ starvation in wild-type and Δ*vtc4* cells are presented on a purine metabolic pathway (A) and a pyrimidine metabolic pathway (B). P_i_-containing substrates are marked as red. Gray indicates enzymes involved in the reaction. Different letters on the graph indicate a significant difference by Tukey-Kramer test (*P* < 0.05). Download FIG S5, TIF file, 0.9 MB. Copyright © 2023 Kim et al. 2023 Kim et al. https://creativecommons.org/licenses/by/4.0/ This content is distributed under the terms of the Creative Commons Attribution 4.0 International license.

10.1128/mbio.00102-23.6 FIG S6 Metabolic changes in nicotinate and nicotinamide pathways under P_i_ starvation in wild-type and Δ*vtc4* cells. Metabolomic data of yeast under P_i_ starvation in wild-type and Δ*vtc4* cells are presented in a metabolic pathway. P_i_-containing substrates are marked as red. Gray indicates enzymes involved in the reaction. Different letters on the graph indicate a significant difference by Tukey-Kramer test (*P* < 0.05). Download FIG S6, TIF file, 0.8 MB. Copyright © 2023 Kim et al. 2023 Kim et al. https://creativecommons.org/licenses/by/4.0/ This content is distributed under the terms of the Creative Commons Attribution 4.0 International license.

10.1128/mbio.00102-23.7 FIG S7 Metabolic changes in glycolysis under P_i_ starvation in wild-type and Δ*vtc4* cells. Metabolomic data of yeast under P_i_ starvation in wild-type and Δ*vtc4* cells are presented in a metabolic pathway. P_i_-containing substrates are marked as red. Gray indicates enzymes involved in the reaction. Different letters on the graph indicate a significant difference by Tukey-Kramer test (*P* < 0.05). Download FIG S7, TIF file, 1.2 MB. Copyright © 2023 Kim et al. 2023 Kim et al. https://creativecommons.org/licenses/by/4.0/ This content is distributed under the terms of the Creative Commons Attribution 4.0 International license.

Δ*vtc4* cells grown on P_i_-replete medium showed numerous metabolic features of P_i_-starved wild-type cells. They did not, however, simply phenocopy a P_i_ starvation response at a reduced scale. Intermediates of tryptophan degradation, such as kynurenine, kynurenic acid, 3-hydroxykynurenine, and the NAD^+^ precursor quinolinic acid, which did not change significantly in P_i_-starved wild-type cells, were increased in Δ*vtc4* cells already under P_i_-replete conditions and increased further (up to 5-fold) after P_i_ starvation (Fig. 8C; Fig. S6). In Δ*vtc4* cells, the later glycolytic intermediates dihydroxyacetone phosphate and phosphoenolpyruvate were more abundant than in wild-type cells in P_i_-replete medium, and 2/3-phosphoglyceric acid underwent a much more pronounced reduction than in wild-type cells after P_i_ starvation (Fig. 8C; Fig. S7). These results indicate that poly(P) synthesis by VTC has a significant effect on the metabolic profile of cells. Poly(P) synthesis dampens the metabolic consequences of P_i_ starvation, which is consistent with its proposed role as a P_i_ reserve, but it also has, so far, unrecognized metabolic functions under P_i_-replete conditions, as indicated by metabolic features of Δ*vtc4* cells that cannot be recapitulated by P_i_ scarcity or P_i_ starvation of wild-type cells.

## DISCUSSION

Our results extend earlier studies of P_i_ starvation (56, 59, 60). In agreement with these studies, we observed reductions in nucleotides and late glycolytic intermediates and increased nucleoside and nucleobase levels. These changes can be explained by simple mass action (60, 61) because they reduce phosphate-containing metabolites under conditions of intracellular P_i_ shortage. By contrast, early TCA cycle metabolites, such as citric acid, isocitric acid, and oxoglutaric acid, strongly increased during P_i_ starvation. Furthermore, oxygen consumption of yeast increases after P_i_ starvation (62). This suggests that mitochondrial respiration may become activated as an alternative mechanism for energy production because it fixes less P_i_ in metabolic intermediates than ATP production based on glycolysis. In line with this, we made the side observation that P_i_ starvation also caused mitochondrial fragmentation (data not shown). Mitochondria fragment in medium favoring respiration, such as nonfermentable carbon sources or glucose-limited medium (63, 64).

Following P_i_ starvation, two P_i_-containing metabolites increased, AICAR and cGMP (Fig. 2B; Fig. S4A in the supplemental material). Little is known about the roles of cGMP in yeast so far (65); however, it provides a potential link to protein kinase A signaling, which is involved in P_i_ homeostasis and signaling through P_i_ transporters in yeast (66–72). cGMP can also inhibit DNA polymerase (73), which might reduce P_i_ consumption by cells and avoid P_i_ depletion during S phase (47). AICAR is an intermediate of *de novo* purine biosynthesis and activates a master regulator of energy homeostasis, AMP-activated protein kinase (AMPK), in mammalian cells (74), but the yeast AMPK Snf1 does not depend on it. Snf1 is activated by ADP (17, 75). After a block in nucleoside synthesis, accumulating AICAR stimulates the PHO transcription pathway by stabilizing the interaction of the responsible transcription factors Pho4 and Pho2 (17). Furthermore, AICAR reduces the activity of mammalian PPIP5 kinase (57), suggesting that it might contribute to the decline of IP_7_ and IP_8_ after P_i_ starvation that also occurs in these cells (76). However, it has remained unknown how AICAR behaves under P_i_ starvation. Our analysis now shows that AICAR accumulates during P_i_ starvation. AICAR accumulation should be favored by the decrease of ADP and ATP levels following P_i_ starvation because these nucleotides exert feedback inhibition on the first step in purine synthesis and thereby on AICAR synthesis (77). Thus, AICAR may promote expression of PHO genes following P_i_ starvation. However, under P_i_ scarcity, when ATP decreases less severely than under P_i_ starvation, AICAR did not increase. This corresponds to the only partial activation of the PHO pathway under P_i_ scarcity. We hence propose that an increase in AICAR may contribute to switching the PHO pathway from partial to full activation when cells transit from P_i_ scarcity to starvation.

Glucose-6-phosphate is used by the pentose phosphate pathway (PPP) to convert NADP^+^ to NADPH, which is essential for cellular redox homeostasis (78, 79). Although NADPH was not detected in our metabolomic analysis, NADP^+^ decreased, and we hence assume that NADPH should decrease as well. NAD^+^, nicotinic acid mononucleotide, and nicotinic acid, which are precursors of NADP^+^, were all reduced by P_i_ starvation, but intermediates of the kynurenine pathway, also known as the *de novo* NAD^+^ synthetic pathway, were significantly accumulated (Fig. S2). These changes may result from the accumulation of AICAR, which stimulates the expression of enzymes involved in the kynurenine pathway (80). The last metabolite of the kynurenine pathway, quinolinic acid, is converted to nicotinic acid mononucleotide by consuming phosphoribosyl pyrophosphate (PRPP). Because PRPP is produced from ribose-5-phosphate, this reaction may be impaired through the decrease in ribose-5-phosphate under P_i_ starvation, favoring the observed accumulation of quinolinic acid. In addition, the PHO pathway directly promotes the catabolism of NAD^+^ by inducing the vacuolar phosphatase Pho8, which removes P_i_ from nicotinic acid mononucleotide and nicotinamide mononucleotide (81). The decrease in the NAD^+^ and NADP^+^ pools is expected to affect intracellular redox homeostasis. This may increase the dependence of cells on the oxidative stress response for surviving P_i_ starvation, which had been previously observed (62). In line with this, cells with perturbed P_i_ and inositol pyrophosphate homeostasis induce the environmental stress response (82, 83).

We observed increased SAM and decreased SAH under P_i_ starvation. SAM is synthesized from methionine and ATP, releasing P_i_ and PP_i_. SAM provides methyl groups for methyl transfer reactions, generating SAH as a byproduct (84). Histone methylation affects global gene expression patterns by changing the structure of chromatin through interactions with various chromatin remodeling factors and transcription regulators (85, 86). The expression of PHO genes is also under the control of histone methylation. Expression of Pho5 and Pho84 is induced in the Δ*set1* mutant, which affects methylation of Lys4 of histone H3 (87, 88). In addition, the methyltransferase Hmt1 promotes expression of several P_i_-responsive genes (89). Thus, the changes of SAM following P_i_ starvation might alter the intracellular methylation status and thereby provide a further route of input for P_i_-dependent gene expression.

An interesting question is how cells distinguish P_i_ scarcity from P_i_ starvation. This is challenging because P_i_ scarcity can be corrected by cells through partial induction of the PHO pathway. The resulting improved capacity for P_i_ scavenging (e.g., through expression of high-affinity P_i_ transporters) apparently allows them to maintain sufficient metabolic performance to support normal growth. Furthermore, positive feedback loops involving Spl2, a small regulator of the P_i_ transporter Pho90, may stably commit cells to activation of the starvation program, even if this reestablishes sufficient intracellular P_i_ supply (20, 51, 52). Nevertheless, the P_i_ starvation program is not launched in a simple all-or-none fashion. Certain genes are activated at different levels of P_i_ shortage, as exemplified by the gene for the high-affinity transporter Pho84, which is activated earlier than the secreted acid phosphatase Pho5 (18–20). Our analyses of the low-P_i_ state, which were performed in cells that induced Pho84 but not Pho5, provide hints on metabolic changes that might be used by cells to distinguish P_i_ scarcity from P_i_ starvation.

**FIG 9:**
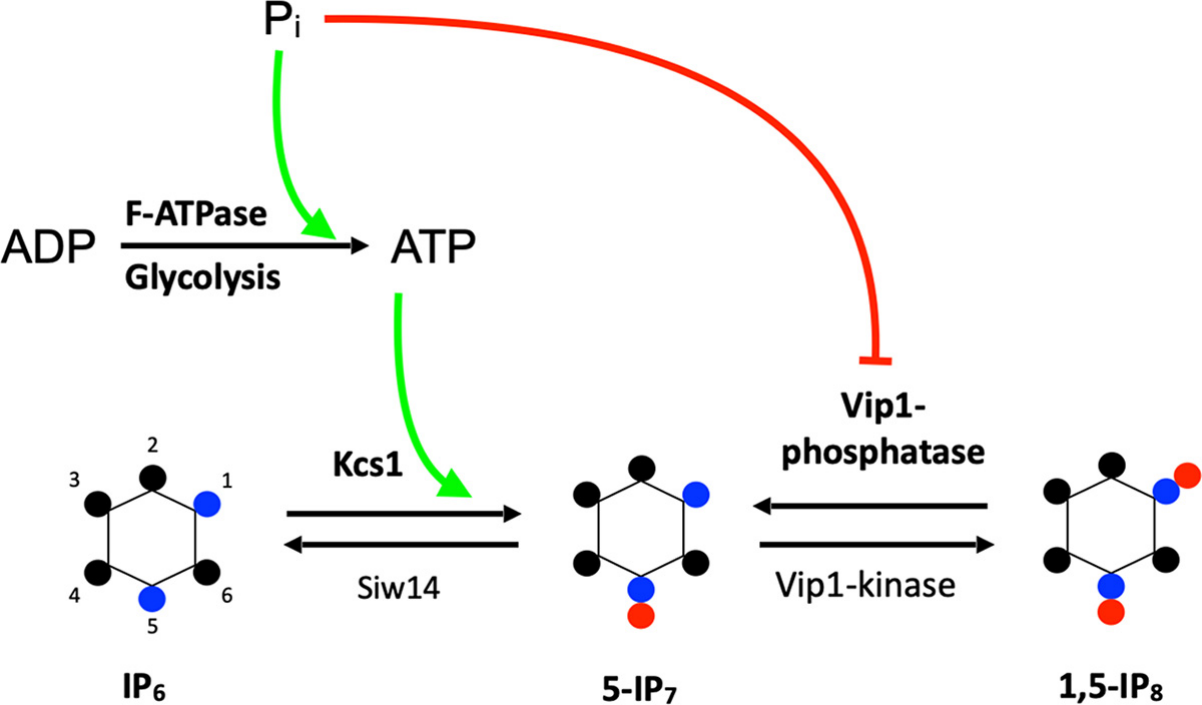
Working hypothesis on the translation of cytosolic P_i_ concentration into changes of PP-IPs. The scheme illustrates the inhibitory (red) and stimulatory (green) influences of P_i_ on key enzymes of ATP and PP-IP production, which are postulated to result from the high *K_m_* and half-maximal inhibitory concentration (IC_50_) values of GAP-DH, F-ATPase, IP_6_ kinase, and PPIP5 kinase. Details are discussed in the main text.

Under P_i_ scarcity, few metabolites changed in a statistically meaningful way, but the levels of ATP, ADP, and PP-IPs decreased by 30 to 50% compared to P_i_-replete conditions. Known properties of the enzymes involved in the production of ATP and in the synthesis of PP-IPs support the following working hypothesis on the reasons for these declines (Fig. 9). Glycolysis and oxidative phosphorylation both contain enzymes that use P_i_ as a substrate, glyceraldehyde-3-phosphate dehydrogenase and F-ATPase, respectively. Both enzymes have *K_m_* values for P_i_ of around 1.5 to 2 mM (90, 91), rendering them susceptible to declines of P_i_ below the 1 to 2 mM threshold that cells normally maintain on P_i_-replete medium. This can reduce ATP production by both pathways following P_i_ scarcity. PP-IP production is sensitive to both ATP and P_i_. The IP_6_ kinases synthesizing inositol pyrophosphates have *K_m_* values for ATP of 1 to 2 mM, which is in the range of the cellular ATP concentration under P_i_-replete conditions (58). While PPIP5 kinase activity, with its *K_m_* for ATP of 0.1 mM, is shielded from changes in ATP and weakly stimulated by P_i_ (92, 93), the opposing phosphatase activity of this bifunctional enzyme is increasingly inhibited in the P_i_ range from 0.1 to 2 mM, covering the normal cellular concentration range (93). The combination of these enzymatic properties may allow an even moderate decrease in P_i_ to significantly reduce PP-IP levels. As we recently observed that the PHO pathway is repressed by PP-IPs through the SPX domain of Pho81 (9), we propose that this decline of PP-IPs following P_i_ scarcity reduces Pho85/Pho80 activity and promotes partial Pho4 nuclear translocation and partial activation of the PHO pathway, allowing cells to maintain normal growth.

Yeast stores P_i_ in the form of polymers in acidocalcisome-like vacuoles. Under P_i_-limiting conditions, polyphosphatases degrade polyphosphate and liberate P_i_, which could potentially be brought back to the cytosol through the vacuolar P_i_ transporter Pho91 (34, 45, 46). In this way, the vacuolar poly(P) pool might buffer the cytosol against sudden drops in P_i_ and delay the onset of the P_i_ starvation response (6, 18). In line with this, our metabolomic analysis revealed exaggerated metabolic changes when the poly(P)-deficient Δ*vtc4* mutant was starved for P_i_. This supports a significant role of acidocalcisome-like vacuoles in P_i_ homeostasis, which may provide the proposed buffer for cytosolic P_i_. Such a buffer is of obvious relevance for an ordered transition into P_i_ starvation and cell cycle arrest (48, 94). Surprisingly, metabolic features of P_i_ starvation were observed in the Δ*vtc4* mutant already under high P_i_ conditions. This phenotype may again reflect the buffering function of poly(P). This function may become important even on high-P_i_ medium because the duplication of all nucleic acids and phospholipids generates a very high need for P_i_ in S phase. It has been proposed that this need can transiently exceed the maximal import capacity of cells, necessitating the vacuolar poly(P) store to cover the deficit (47, 48, 54, 95). In line with this, we observed that the amounts of 1,5-IP_8_, 5-IP_7_, and 1-IP_7_ were reduced in the Δ*vtc4* mutant in high-P_i_ medium (Fig. S8E to H). An explanation of the starvation features of Δ*vtc4* cells from this perspective is, however, only partially satisfactory for our data set because Δ*vtc4* cells on high-P_i_ medium shows numerous, but not all, features associated with P_i_ starvation. The trend for some metabolites was even inversed, such as for adenosine, guanosine, orotic acid, acetyl-CoA, and phosphoenolpyruvate. This suggests that poly(P) may have additional metabolic functions that go beyond those of a P_i_ buffer for the cytosol. There is potential for this because poly(P) has a significant role for the storage of cations, such as for Zn^2+^, Ca^2+^, Mn^2+^, and Mg^2+^ (96–99), and also for cation uptake, as shown for Mg^2+^ (100). Furthermore, poly(P) may affect cellular signaling, influencing the stress response (101–104). Here, it may even have direct impact, such as through polyphosphorylation of lysine residues, which modifies yeast topoisomerase 1 (Tpo1) and nuclear signal recognition factor 1 (Nsr1) (105, 106).

In sum, our observations favor a model where lack of P_i_ induces differential metabolic changes, which together promote the P_i_ starvation response. Beginning P_i_ scarcity could be diagnosed through moderate declines in ATP and inositol pyrophosphates, leading the cells to partially activate P_i_ scavenging systems and maintain normal growth. Profound P_i_ starvation entails numerous additional metabolic changes, such as through AICAR, SAM, and strong reductions in ATP and inositol pyrophosphates. These changes may fully stimulate the transcriptional P_i_ starvation and stress responses in a combinatorial manner. We consider the latter point as an attractive potential solution of the specificity problem that is inherent in the task of measuring a very abundant metabolite. Nuclear magnetic resonance (NMR) studies of yeast found total P_i_ concentrations in the cell to be around 20 mM, of which approximately one-fourth is cytosolic (38, 107). This allows for the estimation of cytosolic P_i_ under P_i_-replete conditions at 5 mM, declining to 1 mM after P_i_ starvation. Given that P_i_ is present in the cytosol in millimolar concentrations, it is difficult to envision that it be “measured” by specific binding to a very low-affinity receptor, which might be susceptible to competition by numerous other compounds. Coincidence detection through a network of P_i_-dependent metabolic reactions could, however, generate such specificity, even for a highly abundant ligand such as P_i_. Therefore, we favor this model of P_i_ detection.

## MATERIALS AND METHODS

### Yeast strains.

The S. cerevisiae strains used in this study are listed in Table 4. Endogenous GFP tagging was performed as described previously (108). The yEGFP-CaURA3 was PCR amplified from the plasmid pKT209 by introducing 40-bp homology before and after the stop codon of the *Pho4* gene. Gene deletion was conducted based on the CRISPR-Cas9 system as described previously (109). The single guide RNA (sgRNA) was cloned into the sgRNA expression vector and cotransformed into yeast cells with hybridized double-stranded oligonucleotides, which contain 40-bp homology of each side before the start codon and after the stop codon of the *Vtc4* gene as the templates for homologous recombination. After transformation, positive colonies were selected by colony PCR and sequencing. PCR primers used for genetic manipulation are listed in Table 5.

**TABLE 4:** Yeast strains used in this study

Strain	Genotype	Source
BY4741	*MATa*; *his3*Δ*1*; *leu2*Δ*0*; *met15*Δ*0*; *ura3*Δ*0*	Euroscarf
BY4741 Pho4-yEGFP	*MATa*; *his3*Δ*1*; *leu2*Δ*0*; *met15*Δ*0*; *ura3*Δ*0*; *Pho4-yEGFP*::*CaURA*	This study
BY4741 *vtc4*Δ	*MATa*; *his3*Δ*1*; *leu2*Δ*0*; *met15*Δ*0*; *ura3*Δ*0*; *vtc4*Δ	This study

**TABLE 5:** Primers used in this study

Name^*a*^	Sequence	Purpose
*Pho4*-yEGFP_f	CTGCCGGTACATCCGTCACCTACAGCAGAACGTGAGCACGggtgacggtgctggttta	*Pho4*-yEGFP tagging
*Pho4*-yEGFP_r	AGTCCGATATGCCCGGAACGTGCTTCCCATTGGTGCACGGtcgatgaattcgagctcg	
*Pho4*-yEGFP_colony_f	CCGCTCGCACGGAAATATTT	
*Pho4*-yEGFP_colony_r	ACTAAGGTATCACCTTCAAAC	
*Vtc4*_guide_f	CGAAGATAACGACTTCGATGGTTTTAGAG	*Vtc4* deletion
*Vtc4*_guide_r	CTAGCTCTAAAACCATCGAAGTCGTTATCTTCGACGT	
*Vtc4*_HR_f	AAATCGGCCAATAAAAGAGCATAACAAGGCAGGAACAGCTATACACAGCGTGTTTTTTTTACTGTATAATTAAGTAATAA	
*Vtc4*_HR_r	TTATTACTTAATTATACAGTAAAAAAAACACGCTGTGTATAGCTGTTCCTGCCTTGTTATGCTCTTTTATTGGCCGATTT	
*Vtc4*_colony_f	GACGGAGAGCTACTGACTTGT	
*Vtc4*_colony_r	TGTGATGGTGACGATGGCATG	
*Taf10*_qRT_f	ATATTCCAGGATCAGGTCTTCCGTAGC	qRT-PCR
*Taf10*_qRT_r	GTAGTCTTCTCATTCTGTTGATGTTGTTGTTG	
*Pho5*_qRT_f	GGGCAACACTTTCCACAGAT	
*Pho5*_qRT_r	CAATTGGAACAACAGCATCG	
*Pho84*_qRT_f	TTCTGCTGCATCTGGTAAGG	
*Pho84*_qRT_r	TCCATGACGTGAGGTAACCA	

a f, forward; r, reverse; HR: homologous recombination.

Synthetic complete (SC) medium was prepared using yeast nitrogen base without phosphate (Formedium, UK). The desired phosphate concentration was adjusted by adding KH_2_PO_4_. The potassium concentration was controlled by adding KCl instead of KH_2_PO_4_.

### Media and cell growth.

All media were prepared with ultrapure, UV-treated water from an ultrapurification system (SG, Germany). SC medium was prepared using yeast nitrogen base without phosphate (Formedium, UK). The phosphate concentration was adjusted by adding KH_2_PO_4_, and the potassium concentration was kept constant by substituting KCl for KH_2_PO_4_.

For assays of growth, acid phosphatase, and malachite green, yeast cells were logarithmically grown overnight in 50 mL of SC medium containing 10 mM P_i_ up to an OD_600_ of 1 (4.6 × 10^7^ cells/mL). Cells were sedimented in a table-top centrifuge (3,000 × *g*) and washed with SC medium containing different concentrations of P_i_. After two washing steps, the OD_600_ was measured, and cells were inoculated in 100-mL Erlenmeyer flasks containing 20 mL of SC medium with the desired P_i_ concentration (OD_600_ = 0.05; 2.3 × 10^6^ cells/mL). Cells were incubated at 30°C with shaking at 210 rpm in a shaking incubator (Climo-shaker ISFL-X, Kühner, Switzerland). To assess growth, the OD_600_ was monitored at different time points (0, 2, 4, 8, and 24 h).

For microscopic analysis, RNA extraction for qRT-PCR, poly(P) measurement, inositol pyrophosphate extraction, and yeast metabolite extraction, yeast cells were prepared in the same manner as described above except that they were transferred into 0 mM P_i_ medium after the washing steps (OD_600_ = 0.2; 9.2 × 10^6^ cells/mL).

### Acid phosphatase assay.

An acid phosphatase assay was performed as previously described (110). Cells were grown in the same manner for growth assays as described above. At each time point, 0.2 OD_600_ units of cells (9.2 × 10^6^ cells) were harvested by centrifugation in a bench-top centrifuge (3,000 × *g*) and resuspended in 250 μL of 0.1 M sodium acetate (pH 4.2) and 250 μL of freshly prepared 9 mg/mL *p*-nitrophenyl phosphate. The mixture was incubated at 37°C for 9 min, and 800 μL of 1.4 M Na_2_CO_3_ was added to stop the reaction. After centrifugation, the OD_420_ was measured from the supernatant as acid phosphatase activity.

### Malachite green assay.

Logarithmically grown cells were inoculated into SC medium containing different concentrations of P_i_ as described above. At different time points (0, 2, 4, 6, and 8 h), 1 mL of cell culture was transferred into a microcentrifuge tube and sedimented by centrifugation at 13,000 × *g* for 1 min. The supernatant was transferred into a new tube and diluted with P_i_-free SC medium to be within a linear range of detection (200-fold dilution for 10 mM samples, 10-fold dilution for 0.5 mM samples, and no dilution for 0 mM samples). Fifty microliters of diluted samples was mixed with 32 μL of 0.1 mM malachite green solution containing 0.35% (mass/vol) of polyvinyl alcohol (molecular mass 85,000 to 124,000 Da) and 43 μL of 4.48 mM ammonium molybdate solution containing 12.5% (vol/vol) H_2_SO_4_. After incubation for 15 min at room temperature, the absorbance was measured at 620 nm on a SpectraMax M3 plate reader (Molecular Devices, USA) in a 96-well clear plate with a flat bottom.

### Fluorescence microscopy.

Cells in the logarithmic phase were inoculated in SC medium and grown as described for the growth assay above. Fluorescence images were obtained with a Nikon Eclipse Ti2/Yokogawa CSU-X1 spinning-disk microscope with two Prime BSI scientific complementary metal oxide semiconductor (sCMOS) cameras (Teledyne Photometrics, USA), a LightHub Ultra laser light (Omicron Laserage, Germany), and an Apo total internal-reflection fluorescence (TIRF) ×100/1.49 oil lens (Nikon, Japan). Experiments were repeated at least three times. Representative images are shown in the figures.

### RNA extraction and qRT-PCR.

Total RNA was extracted from 10 OD_600_ units of yeast cells (4.6 × 10^8^ cells) with RNeasy kits (Qiagen, Germany) according to the manufacturer’s instructions. One microgram of total RNA was used for cDNA synthesis using RevertAid reverse transcriptase (Thermo Fisher Scientific, USA). Gene expression levels were quantitatively monitored using real-time PCR (LightCycler 480, Roche, Switzerland) with SYBR Green I master mix (Roche, Switzerland). Gene expression was normalized by using TATA-binding protein-associated factor *Taf10* transcript as an internal control. Primers used for qRT-PCR are listed in Table 5. The mean and standard deviation values of gene expression were calculated from three biological replicates with three technical replicates.

### Poly(P) measurement.

Poly(P) levels were evaluated from cells using the direct 4′-6-diamidino-2-phenylindole (DAPI) assay (62). Cells were logarithmically grown in P_i_-rich SC medium and transferred to SC medium containing different concentrations of P_i_, as described above. After 8 h of incubation, 0.5 OD_600_ units of cells (2.3 × 10^7^ cells) were harvested by centrifugation in a bench-top centrifuge (3,000 × *g*) and washed with 50 mM HEPES-KOH (pH 7.5). The cell pellet was resuspended in 400 μL of DAPI buffer containing 20 mM HEPES-KOH (pH 6.8), 150 mM KCl, and 10 μM DAPI. After two rounds of freeze-thaw in liquid nitrogen, samples were centrifuged for 2 min at 13,000 rpm. The supernatant was diluted with DAPI buffer (1:20), and 200 μL of diluted sample was transferred to black 96-well polypropylene plates. DAPI fluorescence was measured with a SpectraMax Gemini EM microplate reader (Molecular Devices, USA), with excitation and emission set to 420 nm and 550 nm, respectively (111, 112).

### Extraction of inositol pyrophosphates for quantification.

To extract PP-IPs, 1 mL of cell culture grown in the SC medium containing different P_i_ levels (10 mM, 0.5 mM, and 0 mM) for 8 h was transferred into a microcentrifuge tube and mixed with 100 μL of 11 M perchloric acid. The mixture was snap-frozen in liquid nitrogen, thawed again, and centrifuged (3 min, 13,000 rpm, 4°C). The supernatant was transferred into a new tube. Titanium dioxide (TiO_2_) beads (GL Sciences, Japan) were washed twice with water and 1 M perchloric acid (1.5 mg of beads/sample) and were added to the perchloric acid extract from the cells. The sample with TiO_2_ beads was gently rotated for 15 min at 4°C and centrifuged (1 min, 13,000 rpm, 4°C). The beads were washed twice using 500 μL of 1 M perchloric acid and were resuspended in 300 μL of 3% (vol/vol) NH_4_OH by gentle rotation at room temperature. After centrifugation (1 min, 13,000 rpm), the eluents were transferred into new tubes and completely dried in a SpeedVac (Labogene, Denmark) at 42°C.

For PP-IP extractions (Fig. S8A and B), 4 OD_600_ units of cells (1.38 × 10^8^ cells) were harvested using rapid vacuum filtration through a PTFE membrane filter (1.2 μm; Piper Filter GmbH, Germany) (55). Yeast cells on the PTFE membrane were resuspended in 400 μL of 1 M perchloric acid, and the samples were lysed by vortexing with glass beads (0.25 to 0.5 mm) for 10 min at 4°C. After centrifugation (3 min, 13,000 rpm, 4°C), the cleared supernatant was transferred into a new tube, and 4 mg of prewashed TiO_2_ beads was added. The rest of the analysis was performed as described above.

### Analysis of inositol pyrophosphates.

The amounts of PP-IPs were quantified by capillary electrophoresis coupled to electrospray ionization mass spectrometry (CE-ESI-MS) as described previously (113–115). CE-ESI-MS analysis was performed on an Agilent 7100 CE coupled to a triple quadrupole mass spectrometer (QqQ MS) Agilent 6495c, equPP-IPed with an Agilent Jet Stream (AJS) ESI source. Stable CE-ESI-MS coupling was enabled by a commercial sheath liquid coaxial interface, with an isocratic LC pump constantly delivering the sheath liquid.

All experiments were performed with a bare fused silica capillary with a length of 100 cm and a 50-μm internal diameter (i.d.); 35 mM ammonium acetate titrated by ammonia solution to pH 9.7 was used as background electrolyte (BGE). The sheath liquid was composed of a water:isopropanol (1:1) mixture, with a flow rate of 10 μL/min. Fifteen microliters of IP extracts was spiked with 0.75 μL of isotopic internal standards mixture (2 μM [^13^C_6_]1,5-IP_8_, 10 μM [^13^C_6_]5-IP_7_, 10 μM [^13^C_6_]1-IP_7_, and 40 μM [^13^C_6_]IP_6_). Samples were introduced by applying 10,000 Pa of pressure for 10 s (20 nL). A separation voltage of 30 kV was applied over the capillary, generating a constant CE current at around 19 μA.

The MS source parameter settings were as follows: nebulizer pressure was 8 lb/in^2^, sheath gas temperature was 175°C with a flow rate at 8 L/min, gas temperature was 150°C with a flow rate of 11 L/min, and the capillary voltage was −2,000 V with a nozzle voltage of 2,000 V. Negative high pressure radio frequency and low pressure radio frequency (ion funnel parameters) was 70 V and 40 V, respectively. Parameters for multiple reaction monitoring (MRM) transitions are indicated in Table 6.

**TABLE 6:** Parameters for MRM transitions

Compound	Precursor ion	Product ion	Dwell	CE (V)	Cell acceleration (V)	Polarity
[^13^C_6_]IP_8_	411.9	362.9	80	10	1	Negative
IP_8_	408.9	359.9	80	10	1	Negative
[^13^C_6_]IP_7_	371.9	322.9	80	10	1	Negative
IP_7_	368.9	319.9	80	10	1	Negative
[^13^C_6_]IP_6_	331.9	486.9	80	17	1	Negative
IP_6_	328.9	480.9	80	17	1	Negative

### Preparation of yeast metabolite extracts.

Log-phase yeast cells grown in synthetic complete (SC) medium (0.5 OD_600_ units) were collected by the vacuum filtration method using a PTFE membrane filter (1.2 μm; Piper Filter GmbH, Germany) (55). Yeast cells on PTFE membranes were resuspended with a methanol:water (4:1, vol/vol) mixture. The samples were homogenized with a Cryolys Precellys 24 sample homogenizer (Bertin Technologies, USA) with ceramic beads. Homogenized extracts were centrifuged for 15 min at 4,000 × *g* at 4°C. The precipitated protein pellets were used to measure total protein concentration, and the supernatant was collected and evaporated in a vacuum concentrator (LabConco, USA). Dried sample extracts were resuspended in a methanol:water (4:1, vol/vol) mixture based on the total protein content.

### LC-MS.

Untargeted metabolite profiling was performed by hydrophilic interaction liquid chromatography coupled to tandem mass spectrometry (HILIC-MS/MS) in both positive and negative ionization modes using a 6495 triple quadrupole system (QqQ) interfaced with a 1290 ultrahigh-performance LC (UHPLC) system (Agilent Technologies, USA) (116). In positive mode, the chromatographic separation was performed in an Acquity BEH Amide, 1.7-μm, 100 mm × 2.1 mm i.d. column (Waters, USA). The mobile phase was composed of A (20 mM ammonium formate and 0.1% formic acid in water) and B (0.1% formic acid in acetonitrile). The linear gradient elution from 95% B (0 to 1.5 min) down to 45% B was applied (1.5 to 17 min), and these conditions were held for 2 min. Initial chromatographic conditions were then maintained as a postrun during 5 min for column reequilibration. The flow rate was 400 μL/min, column temperature was 25°C, and the sample injection volume was 2 μL. ESI source conditions were set as follows: dry gas temperature of 290°C, nebulizer of 35 lb/in^2^ and flow of 14 L/min, sheath gas temperature of 350°C and flow of 12 L/min, nozzle voltage of 0 V, and capillary voltage of 2,000 V. Dynamic multiple reaction monitoring (DMRM) was used as acquisition mode with a total cycle time of 600 ms. Optimized collision energies for each metabolite were applied. In negative mode, a SeQuant ZIC-pHILIC (100-mm, 2.1-mm i.d., and 5-μm particle size; Merck, Germany) column was used. The mobile phase was composed of A (20 mM ammonium acetate and 20 mM ammonium hydroxide in water, pH 9.7) and B (100% acetonitrile). The linear gradient elution was from 90% (0 to 1.5 min) to 50% B (8 to 11 min) down to 45% B (12 to 15 min). Finally, the initial chromatographic conditions were established as a postrun during 9 min for column reequilibration. The flow rate was 300 μL/min, the column temperature was 30°C, and the sample injection volume was 2 μL. ESI source conditions were set as follows: dry gas temperature of 290°C and flow of 14 L/min, sheath gas temperature of 350°C, nebulizer of 45 lb/in^2^ and flow of 12 L/min, nozzle voltage of 0 V, and capillary voltage of −2,000 V. DMRM was used as an acquisition mode, with a total cycle time of 600 ms. Optimized collision energies for each metabolite were applied.

### Data preprocessing.

Raw LC-MS/MS data were processed using the Agilent Quantitative analysis software (version B.07.00 MassHunter, Agilent Technologies, USA). Relative quantification of metabolites was based on extracted ion chromatogram (EIC) areas for the monitored MRM transitions. The obtained results were exported to R software (http://cran.r-project.org/), and signal intensity drift correction was done within the LOWESS/Spline normalization program followed by noise filtering (Coefficient of Variance [Quality Control features] of >30%).

### Statistical analysis of metabolite profiling.

Statistical analyses of metabolomic data were performed by MetaboAnalyst 5.0 (https://www.metaboanalyst.ca/) (117). Before analysis, signal intensity data were median normalized, log transformed, and mean centered using the autoscaling method. PLS-DA of the first metabolic profiling with different P_i_ conditions was conducted by considering the P_i_ concentration order. The heatmap of the correlation matrix between metabolites of different P_i_ conditions was calculated by the Pearson *r* correlation coefficient. Volcano plot analysis was performed by a two-sample *t* test. The metabolites showing a *P* value of <0.1 with an absolute value fold change (|FC|) of >1.5 (10 mM P_i_ versus 0.5 mM P_i_) or |FC| of >2 (10 mM P_i_ versus 0 mM P_i_) were considered statistically meaningful metabolites. Results of the volcano plot analysis were exported and visualized with GraphPad Prism 9 (GraphPad Software, USA). For the second metabolic profiling analysis, using wild-type and Δ*vtc4* cells, the prominent outliers from the PLS-DA were removed before further analyses. A two-way ANOVA followed by false discovery rate correction (*P* < 0.05) was performed to investigate metabolite variabilities between two different factors, genotype (wild type and Δ*vtc4*), and P_i_ conditions (10 mM P_i_ and 0 mM P_i_) and their interaction. A hierarchical clustering heatmap was generated using the Euclidean distance measure with Ward’s clustering method. The 50 most significantly changed metabolites according to the ANOVA were selected for visualization.

### Pathway analysis and metabolite set enrichment analysis.

Pathway analysis was performed using MetaboAnalyst 5.0 based on the metabolites that statistically significantly increased or decreased under 0.5 mM P_i_ (|FC| > 1.5; *P* < 0.1) or 0 mM P_i_ (|FC| > 2; *P* < 0.1) conditions compared to under 10 mM P_i_ conditions. Hypergeometric test and relative betweenness centrality were used for the enrichment method and topology analysis, respectively. Metabolite set enrichment analysis was performed by MetaboAnalyst 5.0 based on 84 metabolite sets of KEGG human metabolic pathways. Results of pathway analysis and metabolite set enrichment analysis were exported and visualized with GraphPad Prism 9.
