# Supplementary figures and images for: Metabolic Consequences of Polyphosphate Synthesis and Imminent Phosphate Limitation
Source: mBio. 2023 Apr 19;14(3):e00102-23. doi: 10.1128/mbio.00102-23 (PMC10294617; doi:10.1128/mbio.00102-23)

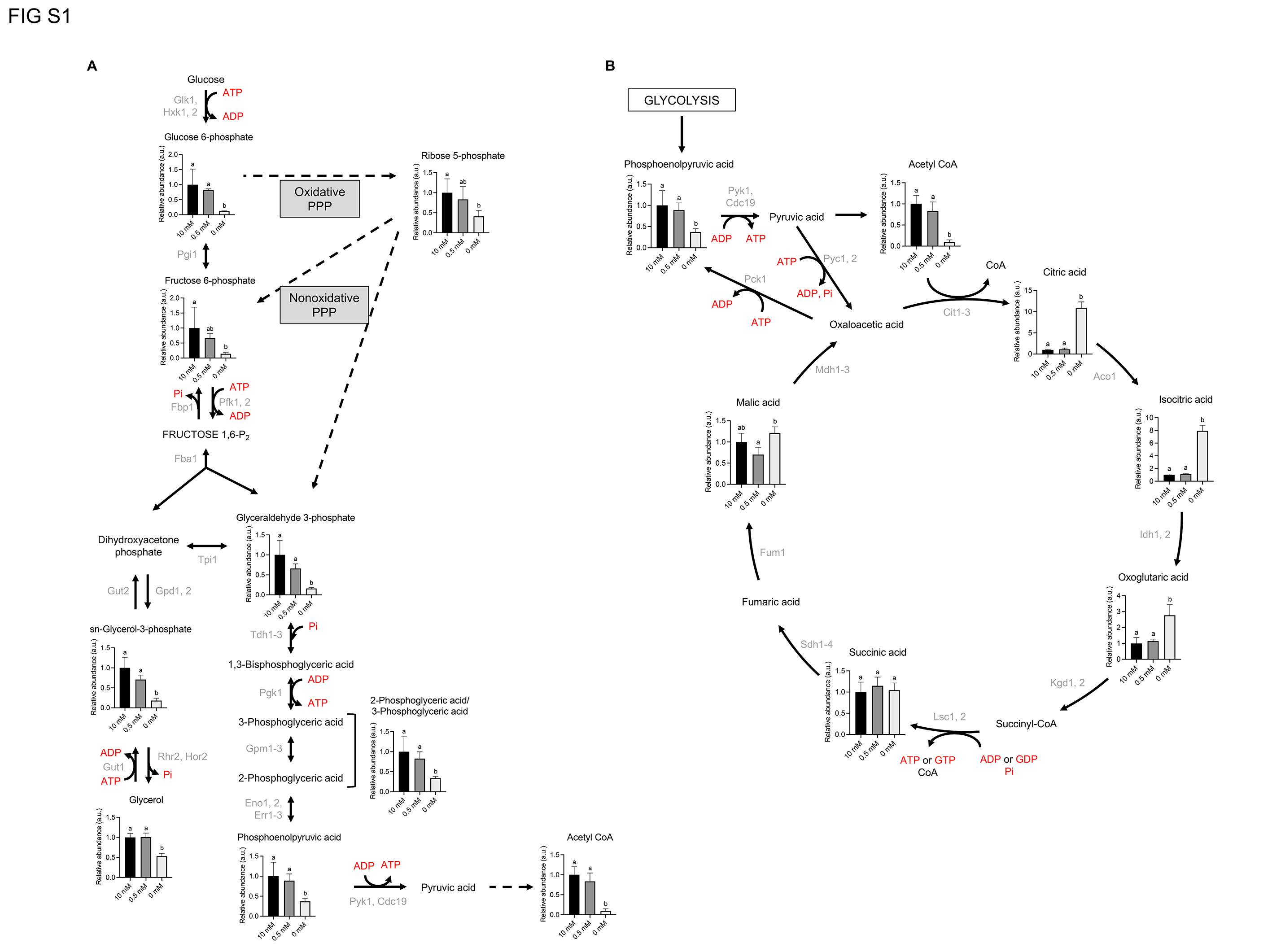

Supplement: FIG S1 [file mbio.00102-23-s0001.tif]

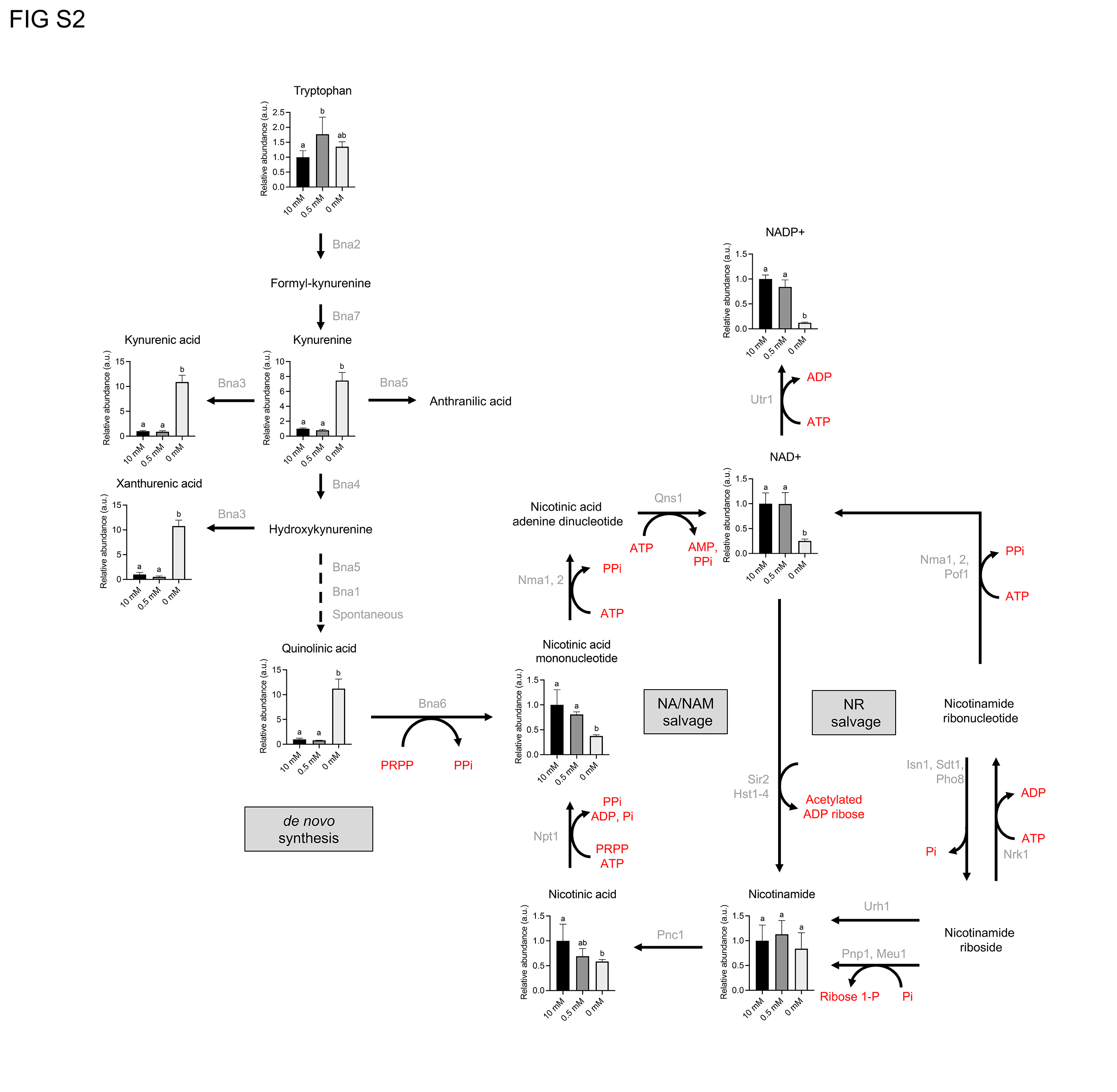

Supplement: FIG S2 [file mbio.00102-23-s0002.tif]

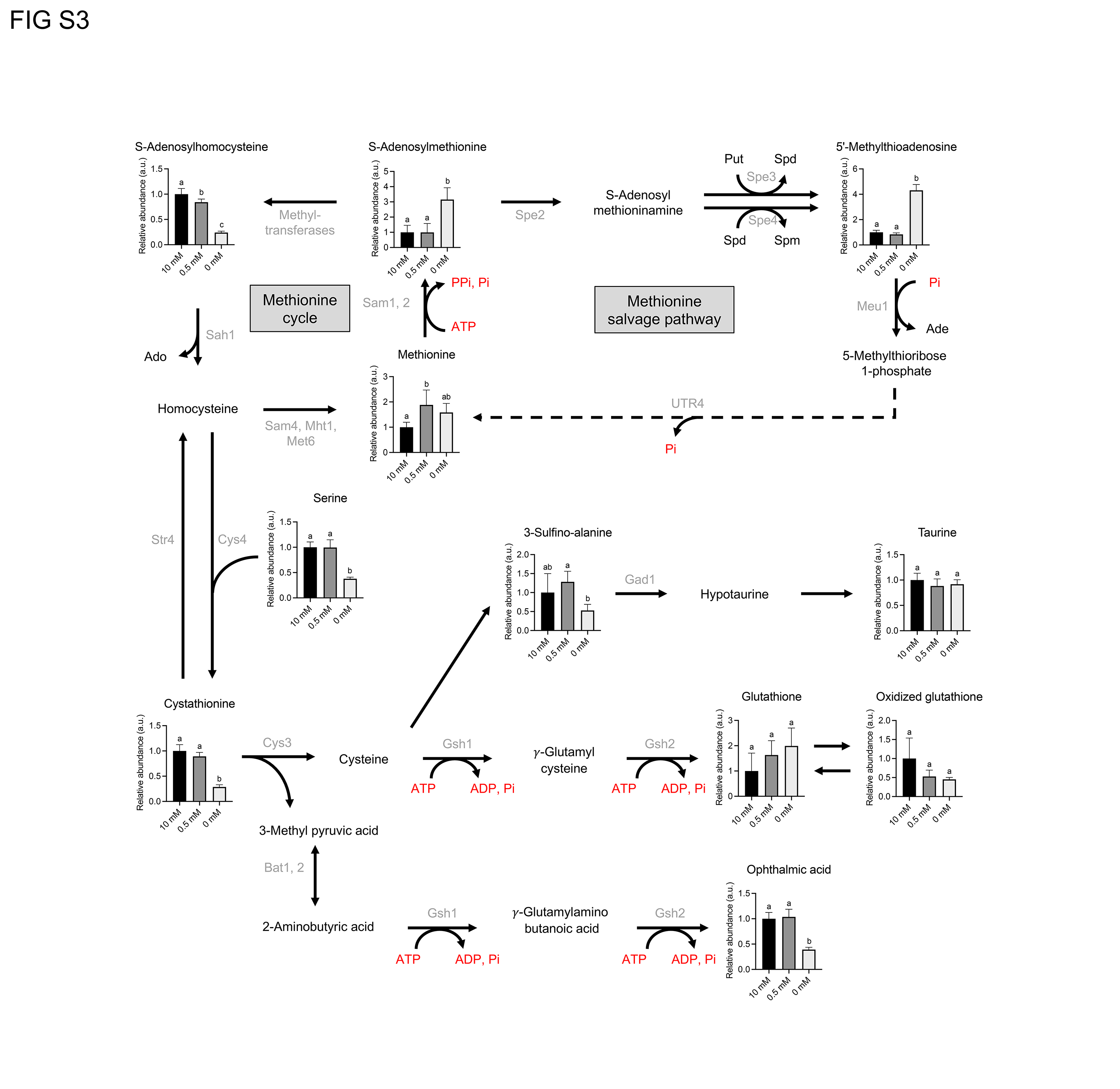

Supplement: FIG S3 [file mbio.00102-23-s0003.tif]

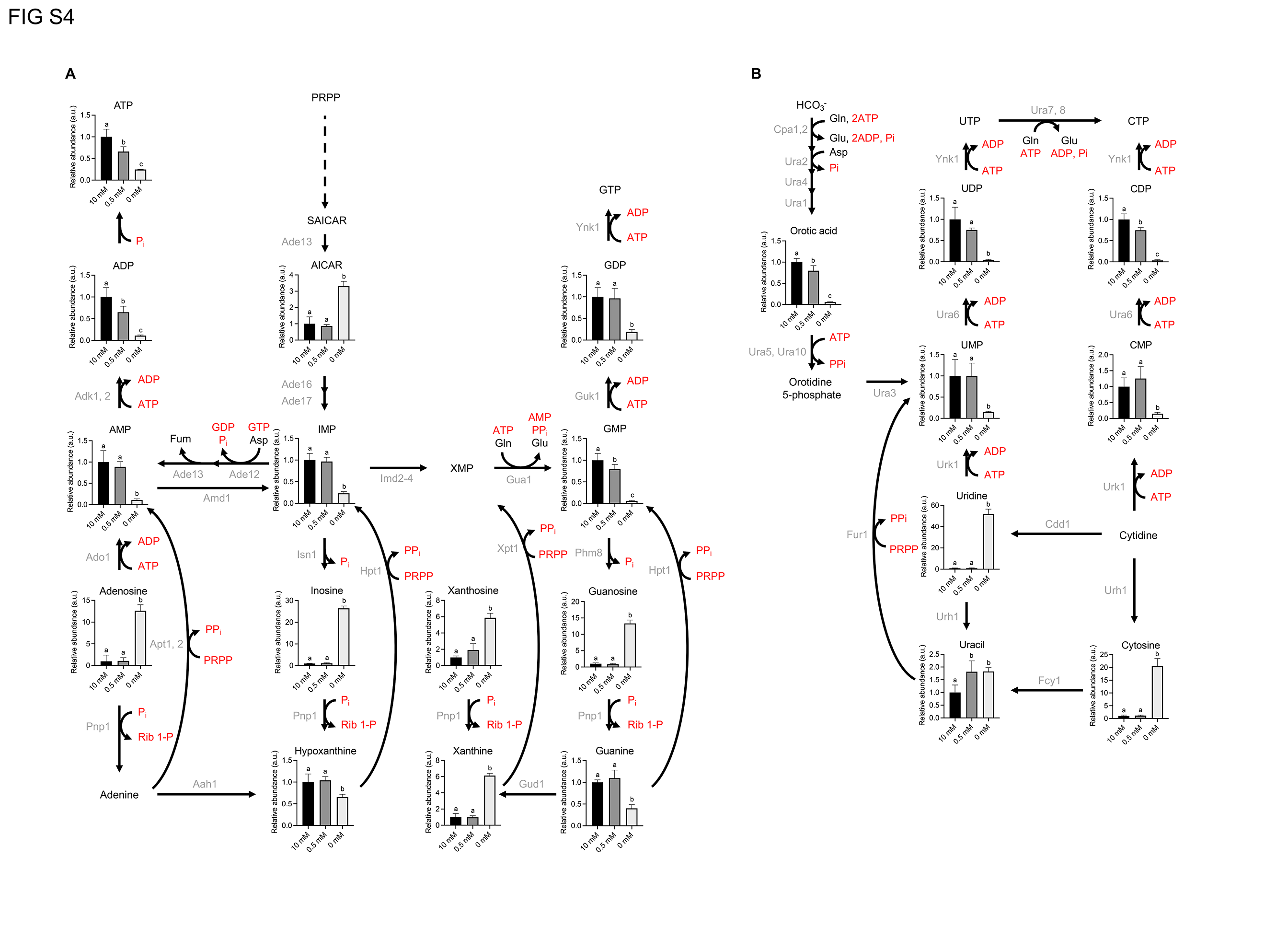

Supplement: FIG S4 [file mbio.00102-23-s0004.tif]

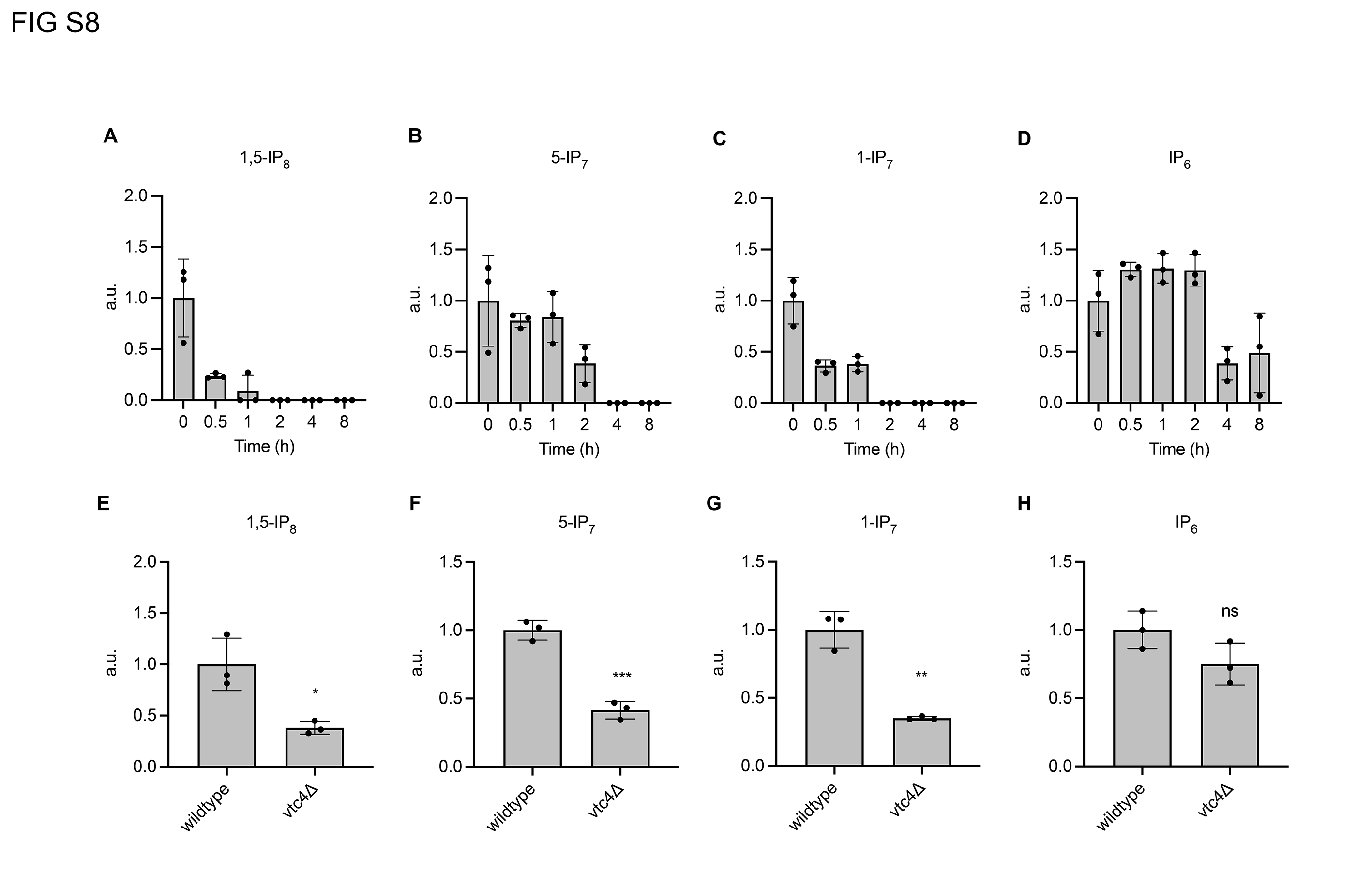

Supplement: FIG S8 [file mbio.00102-23-s0008.tif]

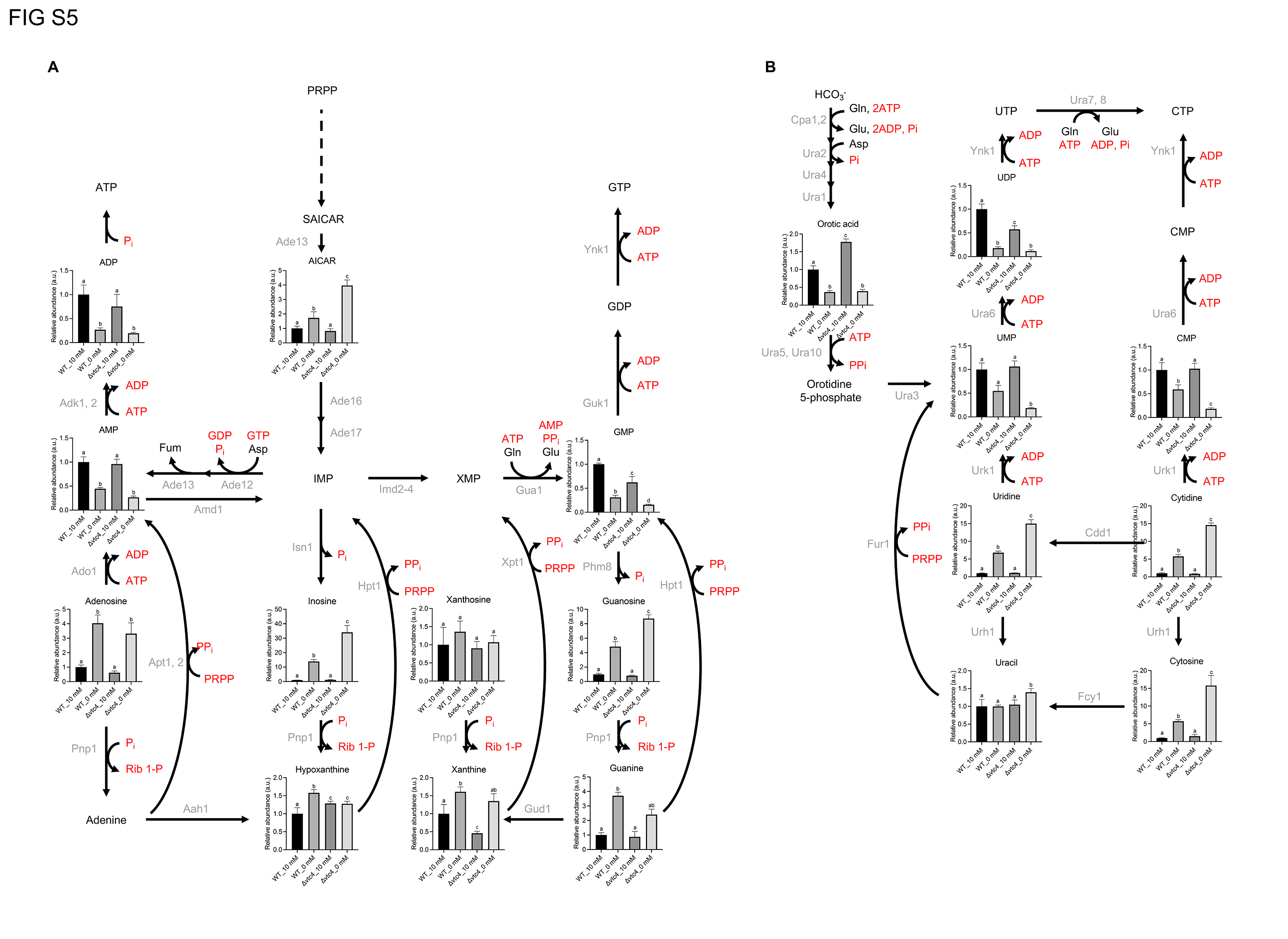

Supplement: FIG S5 [file mbio.00102-23-s0005.tif]

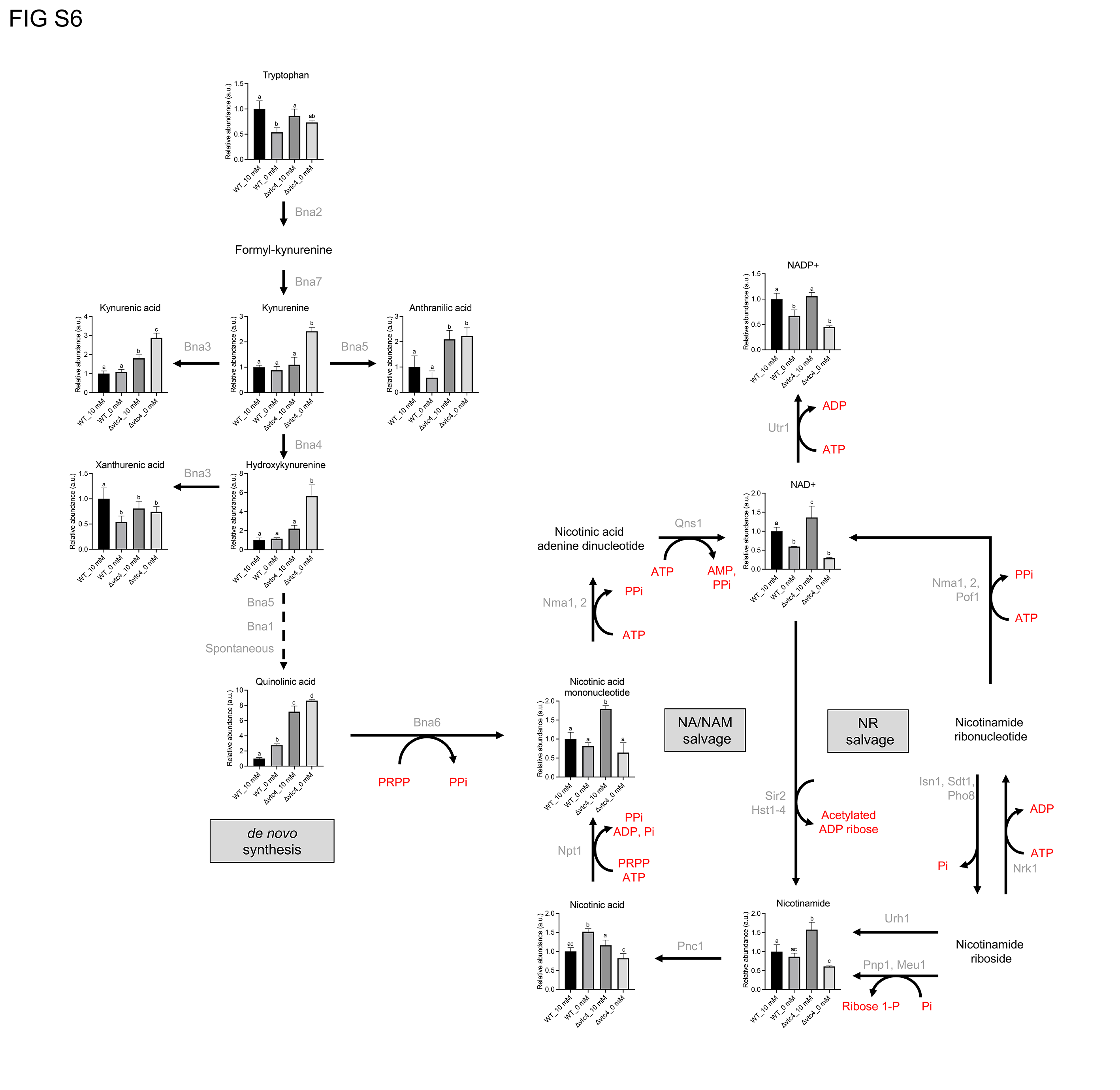

Supplement: FIG S6 [file mbio.00102-23-s0006.tif]

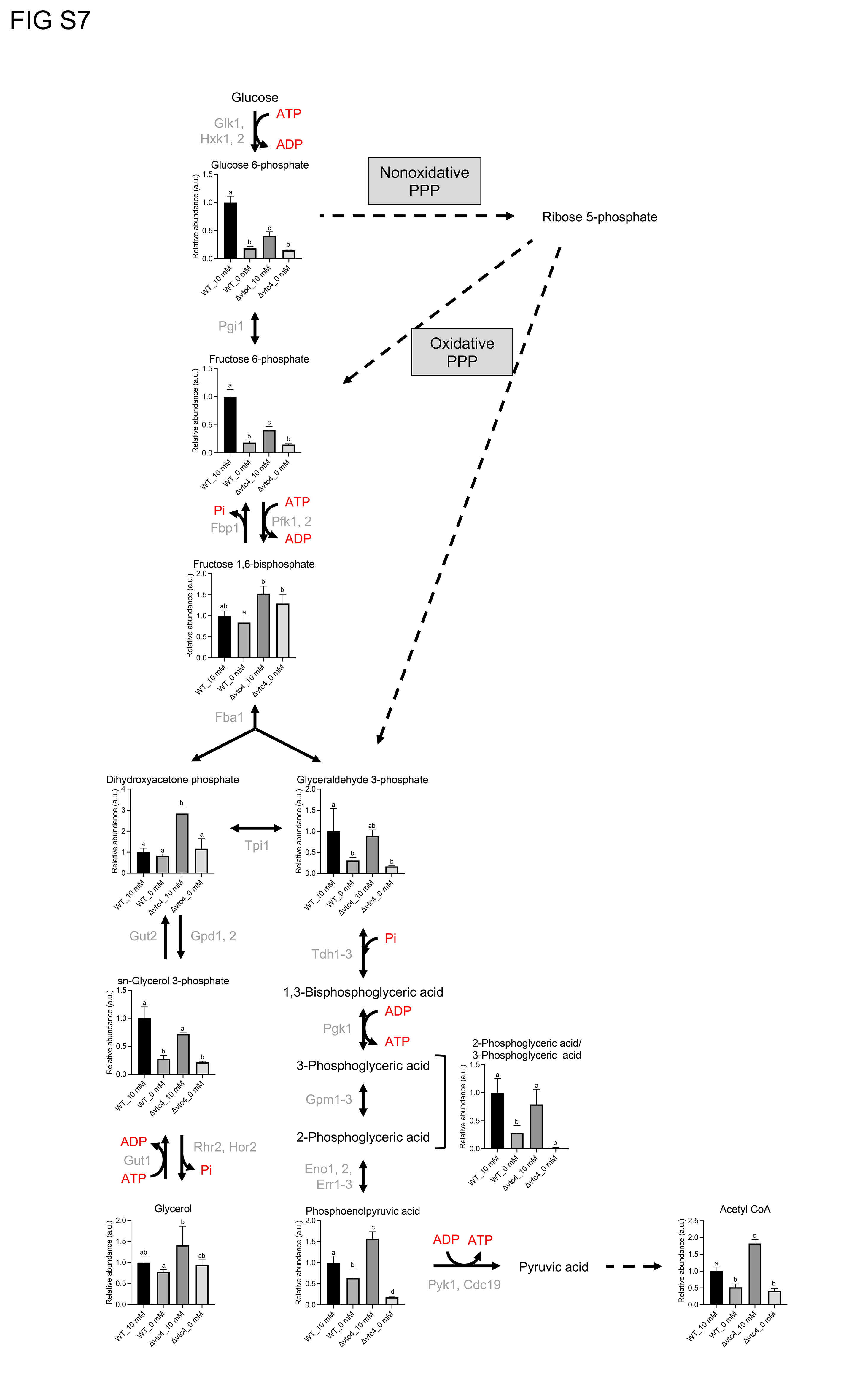

Supplement: FIG S7 [file mbio.00102-23-s0007.tif]
